# On the Structure and Redox Behavior of Ni and Cu Single Atoms Supported on Carbon Nitride

**DOI:** 10.1002/anie.2299555

**Published:** 2026-04-02

**Authors:** Giovanni Colonnello, Ksenija Maver, Arianna Actis, Gaia Grando, Giacomo Filippini, Tiziano Montini, Michele Melchionna, Paolo Fornasiero, Lucia Nasi, Iztok Arčon, Lorenzo Donà, Bartolomeo Civalleri, Enrico Salvadori, Mario Chiesa

**Affiliations:** ^1^ Department of Chemistry University of Torino Torino Italy; ^2^ DII – Department of Industrial Engineering University of Padova Padova Italy; ^3^ Department of Chemical and Pharmaceutical Sciences Center for Energy INSTM Trieste Research Unit University of Trieste Trieste Italy; ^4^ Nanotechnology Centre Centre for Energy and Environmental Technologies VŠB–Technical University of Ostrava Ostrava‐Poruba Czech Republic; ^5^ Institute of Materials for Electronics and Magnetism National Research Council (IMEM‐CNR) Parma Italy; ^6^ University of Nova Gorica Vipavska 13, Nova Gorica Slovenia and Jožef Stefan Institute Jamova 39 Ljubljana Slovenia

**Keywords:** carbon nitride, catalysis, EPR spectroscopy, nickel, single‐atom catalysts

## Abstract

Single‐atom catalysts (SACs) offer molecular‐level control in heterogeneous catalysis, but their activity depends on whether the support can sustain metal‐centered redox cycling. Here, Ni and Cu single atoms on carbon nitride (CN_x_) are compared to determine how coordination geometry governs redox reversibility and photocatalytic performance. EPR/ENDOR spectroscopy, x‐ray absorption, and DFT identify a unique edge MN_4_ site, composed of three sp^2^ nitrogens and one bridging sp^3^ nitrogen, as the binding motif for both metals. While Ni and Cu occupy the same MN_4_ site in the oxidized state, their redox behavior diverges. Ni preserves a distorted square–planar geometry and undergoes fully reversible Ni^2^^+^/Ni^+^ cycling. In contrast, Cu collapses upon reduction to a low‐coordinate Cu^+^ species that cannot be re‐oxidized. This structural mismatch suppresses catalytic turnover. Accordingly, Ni@CN_x_ efficiently promotes photoredox C─N, C─O, and C─S couplings, whereas Cu@CN_x_ remains inactive. Catalytic performance thus depends on redox compatibility within a rigid binding pocket, rather than on metal identity alone.

## Graphical abstract

Magnetic and electronic spectroscopies, combined with DFT, establish the MN_4_ binding site of Ni and Cu single atoms on carbon nitride and elucidate their redox photochemistry. Ni undergoes geometry‐preserving, reversible M^2^^+^/M^+^ cycling, whereas Cu collapses to an irreversible low‐coordinate Cu^+^ state, suppressing photocatalysis. Redox compatibility within a fixed binding pocket emerges as the decisive criterion for single‐atom catalytic activity.

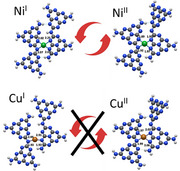

Isolated metal atoms are inherently catalytic [1], yet their reactivity is governed by the first and subsequent coordination spheres provided by molecular ligands or solid supports [2]. Single‐atom catalysts (SACs) exploit this principle by anchoring individual ions in atomically defined environments [3], combining the geometric precision of molecular complexes with the robustness of heterogeneous materials [4]. These systems have become valuable for a number of relevant (photo)catalytic reactions [5, 6, 7, 8], although their performance ultimately hinges on the redox behavior of the embedded metal centers [9].

Carbon nitride (CN_x_), formally built from heptazine and/or triazine units, is widely used as a support, yet its atomic structure depends sensitively on preparation conditions [10, 11], and the nature of the metal binding pockets remains debated. Different sites for metal coordination have been proposed, usually involving nitrogen ligand atoms in idealized heptazine units [12, 13]. However, recent work by Vilè and Pacchioni [14] has shown that in the case of prototypical Ni^II^, the spectroscopic evidences can only be reconciled considering square–planar like sites available at edge boundaries of oligomeric structures. Moreover, essentially no experimental information exists on how these putative sites reorganise upon metal redox cycling.

In redox‐active SACs, the central issue is therefore not only the identity of the binding site but also whether the site can preserve its geometry throughout the catalytic redox cycle and, most importantly, how its geometric rigidity may constrain the electronic configuration of the metal during the redox cycle. This is the key question addressed in the present work.

This concept echoes the entatic‐state model in bioinorganic chemistry, where protein scaffolds pre‐organize the coordination environment to minimize the reorganisation energy associated with metal‐centred redox transformations [15, 16]. Molecular inorganic systems provide analogous examples: subtle ligand‐induced changes in coordination geometry can dramatically shift redox potentials, notably in Cu^II^ complexes where modulation of the N_4_ ligand field governs the accessibility of the Cu^II^/Cu^I^ couple [17]. These examples suggest that the CN_x_ scaffold may enforce distinct redox trajectories on different metals, stabilizing some oxidation states while disfavoring others.

We show that this redox behavior differs markedly for Ni and Cu single atoms supported on CN_x_. Across a series of photoredox model reactions for C─N, C─O, and C─S coupling, relevant to the synthesis of key pharmaceutical, agrochemical, and materials precursors [7, 18, 19, 20], Ni@CN_x_ exhibits robust catalytic activity, whereas Cu@CN_x_ remains largely inactive. Combined experimental and computational analyses reveal that Ni maintains a square–planar MN_4_ environment and undergoes efficient Ni^II^/Ni^I^ cycling, while Cu collapses from a four‐coordinate site to a two‐coordinate Cu^I^ centre that cannot be re‐oxidized. This divergence, rooted in the electronic contrast between Ni^I^ (d^9^) and Cu^I^ (d^10^), directly dictates their opposite photocatalytic performance in cross‐coupling reactions.

To elucidate the geometric and electronic structure of the two SACs, we performed x‐ray absorption spectroscopy (XAS) and electron paramagnetic resonance (EPR) investigations, carrying out both measurements on the very same sample under identical preparation and handling conditions (Supporting Information, sections S1.2 and S1.3). Details on the stepwise synthesis of CN_x_ catalysts are provided in the Supporting Information (Sections S1.1 and S1.4–S1.7). The Ni K‐edge position in as‐prepared Ni@CN_x_ coincides with that of NiO, confirming a +2 oxidation state (Figure 1a, black line), while, under the same conditions, Cu@CN_x_ exhibits a distinct pre‐edge peak at 8982.5 eV, indicative of the partial presence of Cu^I^ species (Figure 1b).

**FIGURE 1:**
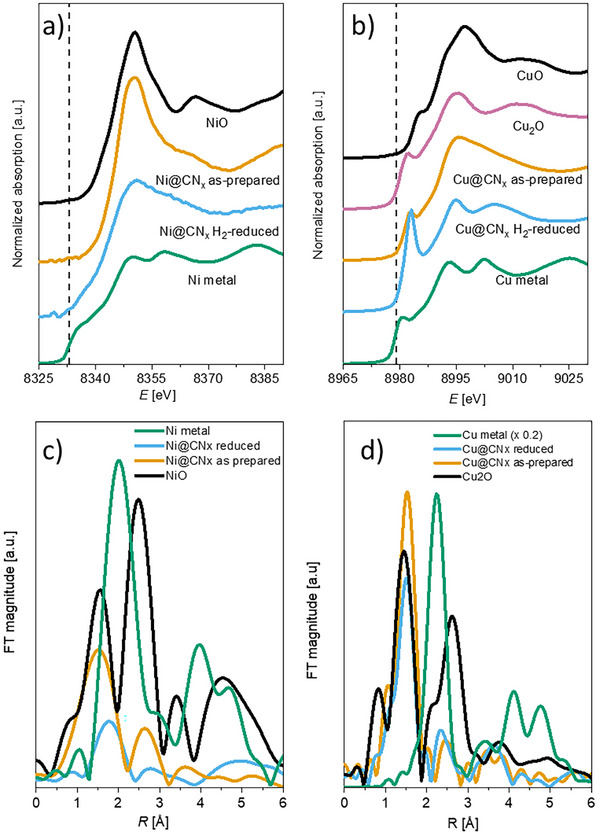
Normalized Ni (a) and Cu (b) K‐edge XANES spectra measured on as‐prepared and reduced Ni@CN_x_ and Cu@CN_x_ catalysts, respectively. For comparison, the spectra of the reference Ni compounds (crystalline NiO as reference for Ni^II^ and Ni metal foil) in (a) and the spectra of the reference Cu compounds (crystalline Cu_2_O as reference for Cu^I^ and Cu metal foil) in (b) are added. The spectra are shifted vertically for clarity. The Fourier transform magnitude of the k‐weighted Ni K‐edge EXAFS spectra and the k^3^‐weighted Cu K‐edge EXAFS spectra of the as‐prepared and the reduced catalyst, calculated in the k range of 3−8 Å^−1^ for Ni and 3−12 Å^−1^ for Cu, are shown in (c) and (d), respectively.

Extended x‐ray absorption fine structure (EXAFS) data (Figure 1c,d; Figures S2 and S4) reveal the absence of metal–metal scattering peaks at distances characteristic of Ni‐Ni (2.95 Å in NiO, Table S3) or Cu─Cu (3.04 Å in Cu_2_O) in both materials, demonstrating that Ni and Cu are atomically dispersed. Consistently, energy‐dispersive x‐ray spectroscopy (EDS) mapping and high‐angle annular dark‐field STEM (HAADF–STEM) show a homogeneous metal distribution with no detectable clustering (Figure S6).

The photocatalytic behavior of Ni@CN_x_ and Cu@CN_x_ SACs with the same metal loading was evaluated under identical conditions using photoredox model reactions of C─N, C─O and C─S coupling between aryl halides and suitable nitrogen‐, oxygen‐, and sulfur‐based molecular precursors. These experiments represented the starting point of our study. The couplings proceed via a dual photoredox mechanism in which visible‐light irradiation (456 nm) of CN_x_ generates photoexcited electrons that are transferred to the metal species (Ni^II^ or Cu^II^) through single‐electron transfer (SET) [7]. The reduced metal then undergoes oxidative addition with the aryl halide, thereby initiating the redox catalytic cycle. Catalytic tests (Supporting Information Section S2, Table S1) comparing two aryl halides (Br and I) revealed that Ni@CN_x_ is highly effective: it promoted C─N coupling in 87%–89% isolated yield (75% after 8 h for iodides) and achieved similarly efficient C─O (70% ± 4%) and C─S (58% ± 9%) formations (Supporting Information Section S2, Table S2). In sharp contrast, Cu@CN_x_ did not afford any cross‐coupled products in any of these reactions, producing only minor dehalogenation by‐products (Supporting Information Section S2, Tables S1 and S2).

Such large differences in catalytic activity cannot originate from textural effects [21], as the two materials exhibit comparable surface areas and porosity, as confirmed by N_2_ physisorption (Figure S5). Moreover, since homogeneous Cu catalysts readily promote C─N couplings [22, 23, 24], the divergent behavior observed here shows that the determining factor is not the intrinsic reactivity of Cu versus Ni, but rather how their redox chemistry is constrained within the CN_x_ scaffold. This raises a central mechanistic issue, namely that two closely related d‐block ions with the same oxidation states give rise to entirely different catalytic outcomes when anchored on CN_x_.

To address this question, we disentangled the intrinsic redox response of Ni and Cu from the complexities of the photocatalytic environment by probing the Ni^II^/Ni^I^ and Cu^II^/Cu^I^ couples under idealized solid–gas conditions. Using EPR, ENDOR, and XAS, supported by DFT, we monitored the redox transitions generated through H_2_/O_2_ cycling and selectively interrogated the paramagnetic intermediates (Ni^I^ and Cu^II^). This approach enabled a direct comparison of the efficiency and reversibility of the two redox couples, and of the geometric rearrangements accompanying reduction.

XAS measurements at the Ni and Cu K‐edges (Figure 1) showed that as‐prepared Ni@CN_x_ contains exclusively Ni^II^, with an edge position matching that of NiO. After H_2_ reduction, the Ni K‐edge shifts by ∼1 eV to lower energy, consistent with formation of Ni^I^, while a pre‐edge shoulder at ∼8335 eV, diagnostic of square–planar coordination, remains in both oxidation states [25, 26]. Cu@CN_x_ displays a markedly different behavior: the pre‐edge feature at 8982.5 eV, associated with Cu^I^, increases substantially upon reduction, indicating full conversion to Cu^I^. EXAFS analysis further shows that reduced Cu@CN_x_ adopts a two‐coordinate, nearly linear Cu^I^ N_2_ first‐shell geometry, in line with the preference of the closed‐shell 3d^10^ configuration for low coordination numbers (Supporting Information section S1.2).

EPR spectroscopy was used to follow redox cycling and probe—in a highly selective way—the local coordination of the paramagnetic intermediates, exploiting the 3d^9^ configuration of Ni^I^ and Cu^II^. Cu^II^@CN_x_ (0.06 wt%, ICP‐OES) exhibits the expected axial *S* = ½ spectrum, with *g*_∥_ > *g*_⊥_ > *g*_e_ and well‐resolved ^63/65^Cu (*I* = 3/2) hyperfine splitting, consistent with a d_x2–y2_ SOMO (Figure 2a). The high‐field region reveals additional ligand hyperfine couplings, which based on ENDOR data correspond to two pairs of equivalent ^14^N nuclei (*I* = 1) with |*A*_z_| ≈ 45 and 51 MHz (Table 1, Figure 3). These couplings indicate coordination to four nearly equivalent N donors in a square–planar environment, analogous to porphyrinic Cu^II^ complexes [27].

**FIGURE 2:**
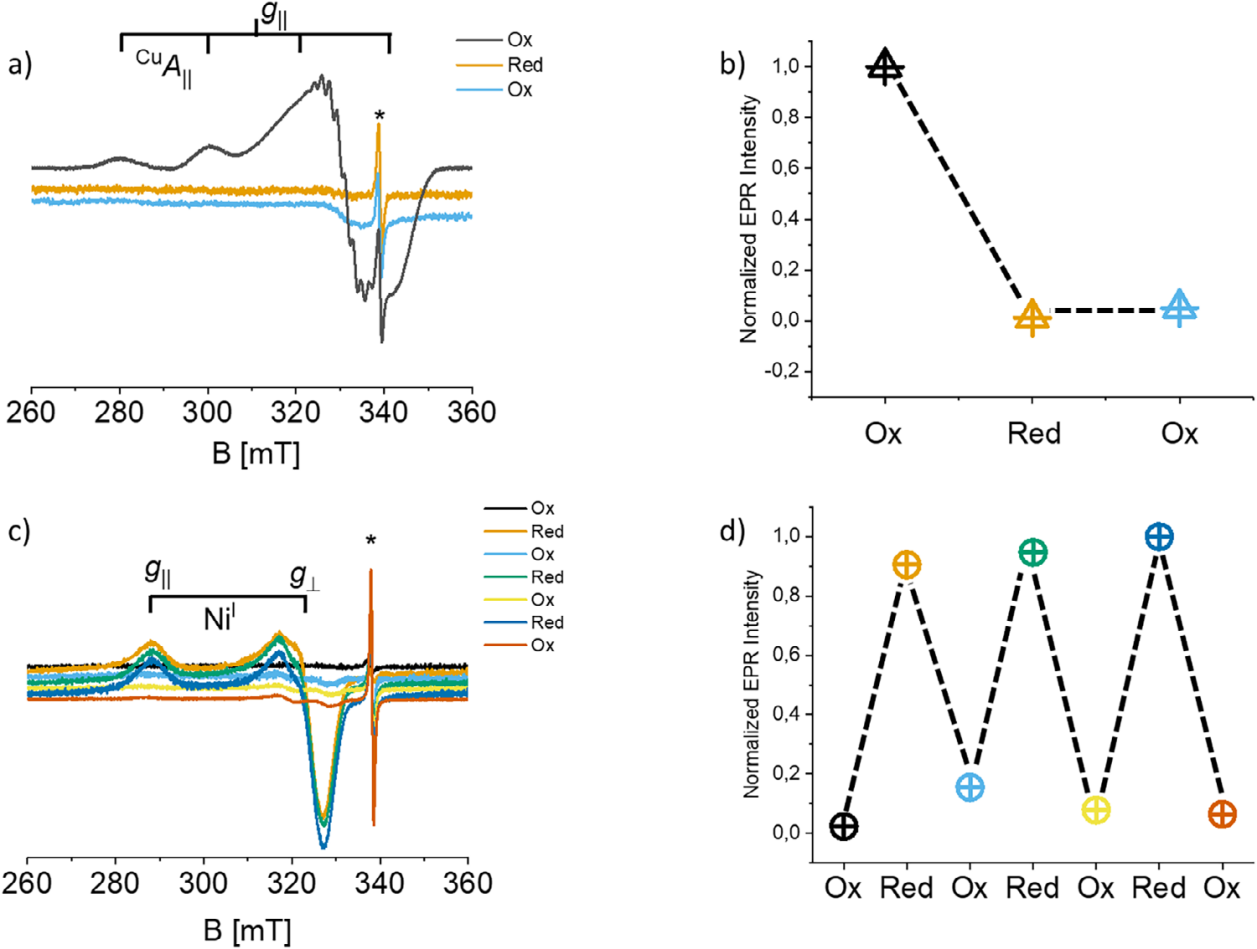
(a) X‐band CW‐EPR spectra of Ni@CN_x_ recorded after oxidizing or reducing treatments as noted; (b) Double integrated intensity of the EPR spectra presented in panel (a); (c) X‐band CW‐EPR spectra of Cu@CN_x_ recorded after oxidizing or reducing treatments as noted; (d) corresponding integrated intensities. All spectra were recorded at 77K. The asterisk indicates a CN_x_ defect.

**TABLE 1:** Spin‐Hamiltonian parameters for Ni^I^ and Cu^II^@CN_x_ catalysts and molecular analogues. Hyperfine constants are given in units of MHz.

	*g*_x_	*g*_y_	*g*_z_	^M^ *A*_x_	^M^ *A*_y_	^M^ *A*_z_	^N^ *A*_x_	^N^ *A*_y_	^N^ *A*_z_^[^ ^c^ ^]^	ref
Ni@CN_x_	2.074 ± 0.012^[^ ^a^ ^]^	2.095 ± 0.013^[^ ^a^ ^]^	2.355 ± 0.04^[^ ^a^ ^]^				11 ± 1 19 ± 1 23 ± 1 27 ± 1	11 ± 1 19 ± 1 23 ± 1 27 ± 1	14 ± 1 22 ± 1 26 ± 1 30 ± 1	This work
	2.066 ± 0.024^[^ ^b^ ^]^	2.120 ± 0.035^[^ ^b^ ^]^	2.370 ± 0.06^[^ ^b^ ^]^							
Ni@CN	2.059	2.114	2.339							[29]
Ni^I^F430 ^[^ ^d^ ^]^	2.063	2.063	2.244				22.0 26.5	26.5 27.0	31.0 34.0	[30]
MCRred1^[^ ^e^ ^]^	2.060	2.070	2.2485				22.5 27.0	25.0 30.6	31.5 36.0	[30]
MCRox2 ^[^ ^e^ ^]^	2.125	2.140	2.227							[31]
MCRred2 ^[^ ^e^ ^]^	2.1753	2.2313	2.2869				11.8 21.0 24.0	13.5 20.2 23.2	16.0 26.2 26.6	[31]
Cu@CN_x_	2.052 ± 0.01	2.052 ± 0.01	2.205 ± 0.001	50 ± 5	50 ± 5	550 ± 5	35 ± 3 44 ± 3	30 ± 3 40 ± 3	45 ± 3 51 ± 3	This work
CuPc ^[^ ^f^ ^]^	2.039 ± 0.01	2.039 ± 0.01	2.157 ± 0.01	83 ± 5	83 ± 5	648 ± 5	44.7 ± 0.5	45.4 ± 0.5	56.5 ± 0.5	[32]
CuTPP ^[^ ^f^ ^]^	2.045	2.045	2.190	102.7 ± 3	102.7 ± 3	615 ± 3	42.8	44.06	54.2	[33]

^a^ Abundance = 36%.

^b^ Abundance = 64%.

^c^ The ^N^ *A*_z_ component is oriented along the *g*_x,y_ axes and lies in the equatorial plane of the molecule.

^d^ Ni(I) Form of Cofactor F4301 of Methanobacterium thermoautotrophicum.

^e^ Ni(I) in Methyl‐coenzyme M reductase.

^f^ Cu(II) phthalocyanine.

^g^ Cu(II) tetraphenylporphyrin.

**FIGURE 3:**
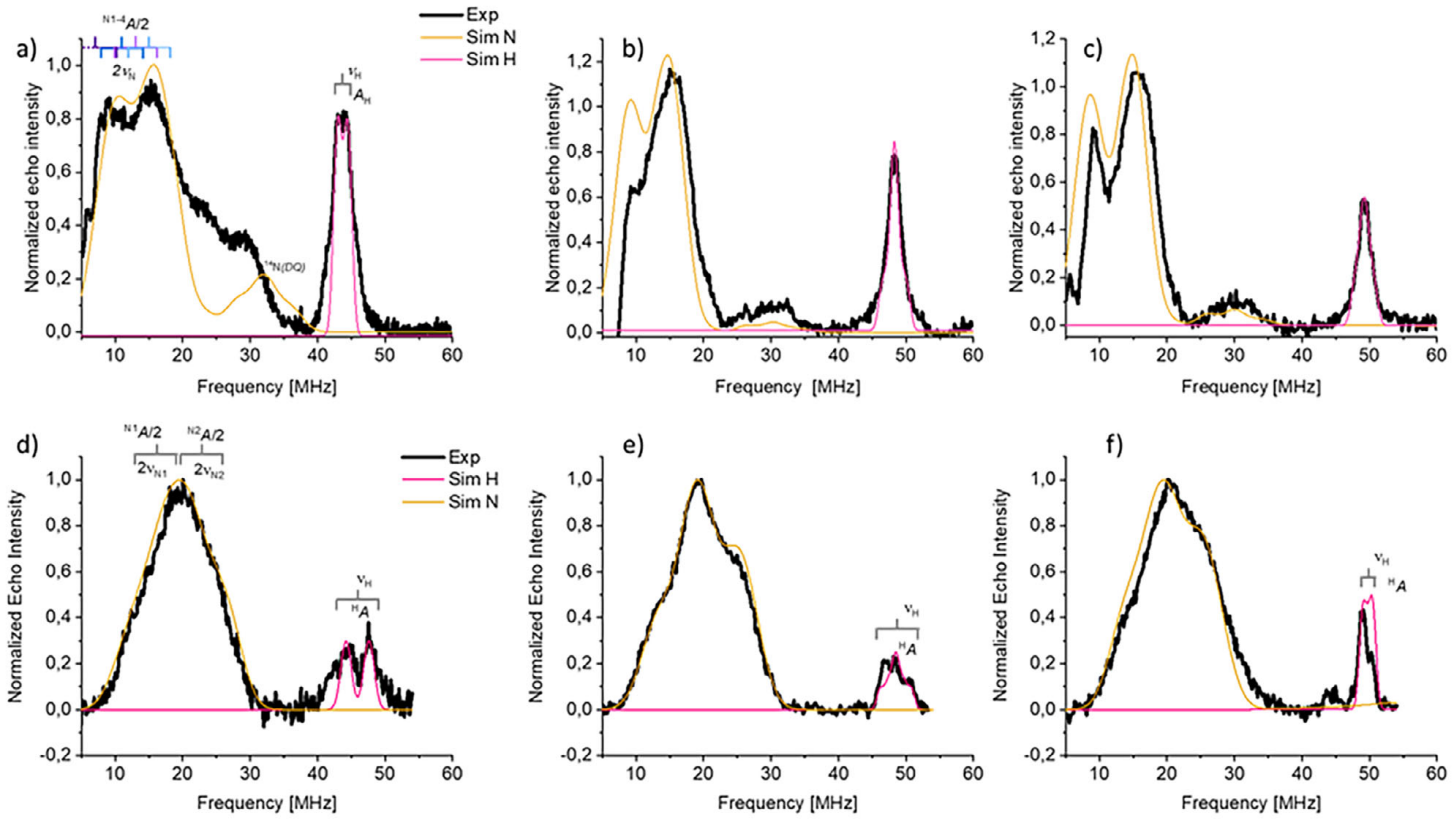
(a)–(c) Experimental and simulated Q‐band (*ν* = 33.83104 GHz) EDNMR spectra of Ni^I^@CN_x_ recorded at **B** = 1026.6 mT (a), **B** = 1134.0 mT (b), **B** = 1156.3 mT (c); (d)–(f) Experimental and simulated Q‐band (*ν* = 33.82373 GHz) Davies ENDOR spectra of Cu^II^@CN_x_ recorded at **B** = 1080.0 mT (d), **B** = 1140.0 mT (e), **B** = 1170.0 mT. All spectra were recorded at 10 K. Simulation of the ^14^N nuclei is in yellow, while the simulation of the ^1^H interaction is shown in pink.

Quantitative EPR indicates that ∼60% of Cu is initially present as Cu^II^, indicating partial reduction to EPR‐silent Cu^I^, in accordance with XAS data (Figure 1b). Increasing Cu loading up to 1.2 wt% (Figure S16) shows no significant changes in EPR‐active Cu^II^, suggesting a constant number of isolated Cu^II^ single‐atom sites (∼1 × 10^1^ ^9^ sites g^−^^1^).

After reduction under H_2_, the Cu^II^ signal vanishes and is not restored upon reoxidation (Figure 2b), demonstrating irreversible collapse of Cu^II^→Cu^I^ within the CN_x_ pocket. By contrast, Ni^II^@CN_x_ is EPR‐silent due to its low‐spin d^8^ configuration, but reduction generates a strongly anisotropic Ni^I^ (3d^9^) EPR signal (Figure 2c), characteristic of a $d_{x2ytext2}$ SOMO [28], and quantitative EPR shows that ∼85% of Ni participates in the Ni^II^/Ni^I^ transition. Crucially, the Ni^I^ signal is fully restored upon oxidation, and repeated redox cycling is possible without loss of intensity (Figure 2d). XRD patterns recorded prior and after the redox cycles are superimposable (Figure S7) demonstrating that the structure of the catalyst is preserved.

EPR measurements performed on catalysts recovered after reaction showed that Ni@CN_x_ retains its redox activity, reproducing the spectroscopic signatures of reversible Ni^I^/Ni^II^ cycling and demonstrating that the Ni centers undergo sustained redox transitions under operating conditions. In contrast, Cu@CN_x_ displayed no regeneration of Cu^II^ after catalysis, consistent with the formation of a stabilized, low‐coordinate Cu^I^ species that cannot be re‐oxidized (Figures S17 and S18). Importantly, x‐ray absorption measurements on the same spent catalysts fully corroborate this picture: XANES/EXAFS spectra of spent Ni@CN_x_ closely resemble those of the as‐prepared material, confirming the persistence of Ni^II^ in a four‐coordinate N_4_ environment (Figure S29, Table S5), whereas spent Cu@CN_x_ is dominated by Cu^I^ species and exhibits an EXAFS signature compatible with two‐coordinate N_2_ binding (Figure S30, Table S5). The convergence of EPR and XAS evidence demonstrates that Ni@CN_x_ preserves a four‐coordinate environment and fully reversible Ni^I^/Ni^II^ redox cycling under catalytic conditions, whereas the Cu coordination sphere undergoes irreversible collapse to Cu^I^; this intrinsic divergence directly accounts for the inactivity of Cu‐based SACs and the sustained performance of Ni@CN_x_ across C–N, C–O, and C–S photoredox couplings.

To elucidate the local coordination of Ni^I^ and Cu^II^, we employed hyperfine spectroscopies. In contrast to x‐ray techniques, which yield coordination numbers and distances averaged over all metal sites, hyperfine methods provide element‐ and oxidation‐state–selective information and are exquisitely sensitive to the immediate ligand environment. This makes them particularly suited to resolving the local geometry of the paramagnetic Ni^I^ and Cu^II^ centers. The CN_x_ binding site, composed of nitrogen donor atoms, gives rise to hyperfine interactions dependent on coordination geometry, though these are often unresolved in CW‐EPR. High‐resolution hyperfine techniques recover NMR transitions of nuclei coupled to the electron spin. Figure 3a–c shows orientationally selective EDNMR spectra of Ni^I^, revealing a strong absorption between 9–40 MHz from strongly coupled ^14^N nuclei (*A* > 2ν_I_), and a proton peak at ca. 50 MHz. These spectra were simulated at once with complementary X‐band Matched HYSCORE and Q‐band Davies ENDOR experiments (Figure S19) to derive a single set of parameters (Table 1). The broad signals are attributed to four non‐equivalent ^14^N nuclei with couplings from 14–30 MHz, as confirmed by cross‐peaks in the (‐,+) HYSCORE quadrant (Figure S19b). Similarly, Q‐band Davies ENDOR was used to characterize the ligand nitrogens in Cu^II^@CN_x_. The spectra (Figure 3d–f) show broad lines, modelled with two ^14^N couplings (Table 1), consistent with Cu^II^ porphyrin‐like systems [32, 33]. For both Ni^I^ and Cu^II^, a proton coupling (∼10 MHz) is observed, indicating a metal–proton distance of ∼0.35 nm.

Thus, although Ni and Cu reside in similar MN_4_ environments, only Ni undergoes reversible M^II^/M^I^ cycling within CN_x_, whereas Cu becomes irreversibly trapped in a reduced state.

These redox behaviors rationalise the catalytic outcomes directly. Ni preserves a four‐coordinate geometry upon reduction, enabling efficient Ni^II^/Ni^I^ cycling under photoredox conditions, whereas Cu^I^, constrained by the rigid CN_x_ framework, relaxes into a linear two‐coordinate configuration incompatible with reoxidation. The catalytic experiments therefore illustrate the operational consequences of the underlying geometric constraints.

In order to translate the spectroscopic evidence in atomistic models, we employed DFT calculations aimed at modelling the binding site of the two metals in both oxidation states. Based on the constraints emerging from the EPR data, we consider structures that provide four coordinating nitrogen ligands either at the edges of polymeric structures or through unreacted heptazine molecules [14]. In Figure 4, we present the structures (namely 3sp2_sp3) which show the best agreement with the experimental findings, while the other structural models are shown in Figures S20–S28.

**FIGURE 4:**
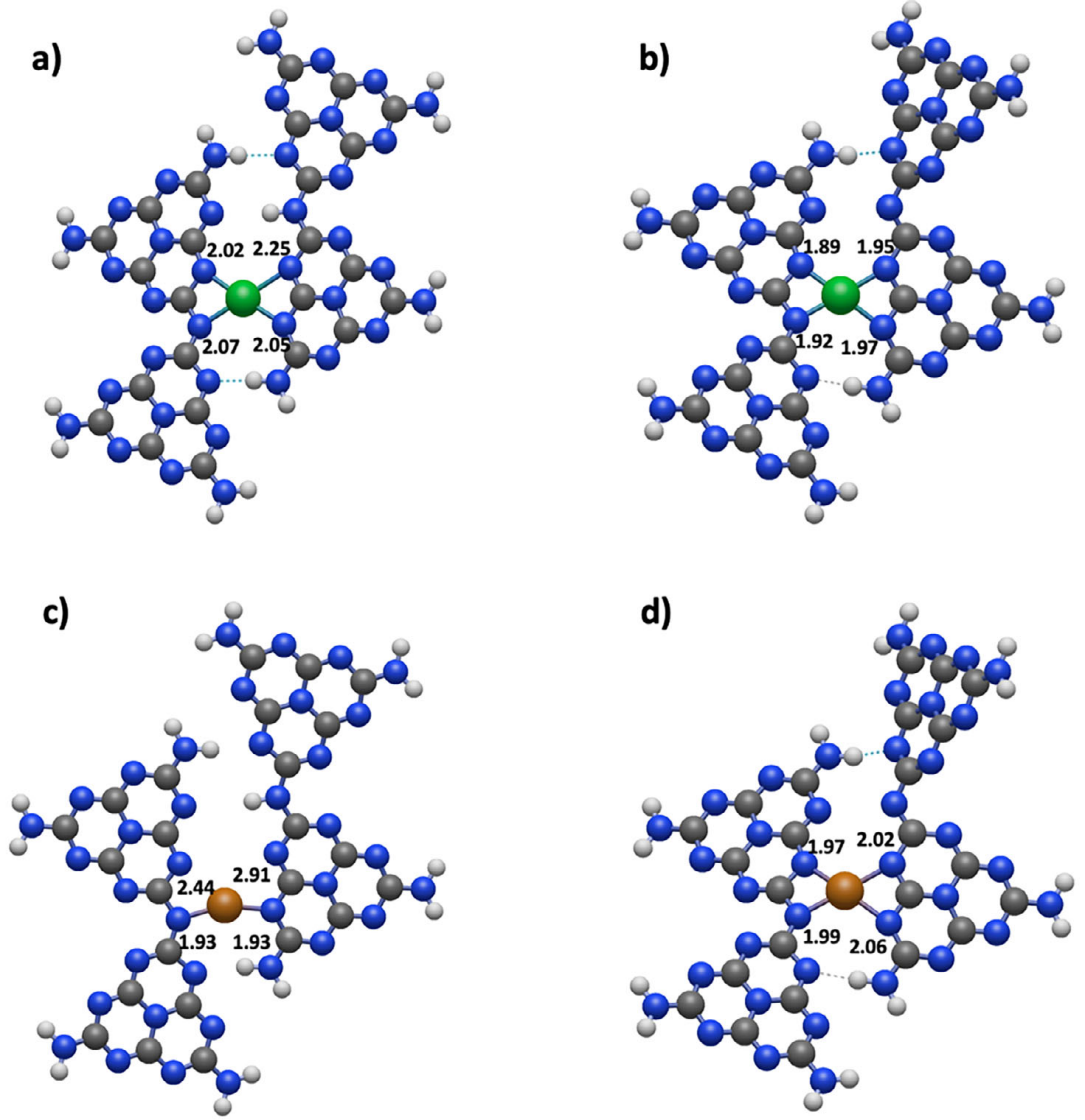
Optimized DFT structures for the proposed 3sp [2]_sp [3] tetramelem models for Ni^I^ (a), Ni^II^ (b), Cu^I^ (c) and Cu^II^ (d), respectively. Computed distances between the N atoms of the melem ligands and the metal center are also reported (Å). Color code for atoms: hydrogen in white, carbon in dark gray, nitrogen in blue, nickel in green and copper in brown.

In their +2 oxidation state, both Ni and Cu structures display a quasi‐planar arrangement of the metal coordinated by four nearly equivalent N nuclei with a dihedral angle of 161° < Φ <179° (see Supporting Information for further details) and distances Ni─N in the range 1.89–1.97 Å and Cu─N in the range 1.97 ‐ 2.06 Å (Figure 4b,d). There are two larger N─M─N angles ranging from 111.3° to 117.3° and two smaller spanning from 62.6° to 68.4°, respectively. While this structure is preserved for reduced Ni^I^ (with elongated Ni─N bond in the range of 2.02 ‐ 2.25 Å (Figure 4a)), the coordination of Cu^I^ changes significantly, resulting in two short (1.93 Å and 1.93 Å) and two long (2.44 Å and 2.91 Å) Cu─N bonds, enforcing a two‐coordinate quasi‐linear geometry with a N─Cu─N angle of 157.5° (Figure 4c).

We focus on the magnetic interactions of Ni^I^ and Cu^II^ with the surrounding magnetic nuclei ^14^N and ^1^H (hyperfine interaction). The computed hyperfine parameters are listed in Supporting Information (Figures S20–S28) for the structures investigated in this work. In the case of Ni^I^, one key feature emerging from the experiment is the presence of four inequivalent nitrogen ligands and a proton at a distance of approximately 0.35 nm. The optimised MN_4_ model reported in Figure 4a reproduces the spectroscopic evidence, with three of the four coordinating nitrogen ligands—with formally sp^2^ hybridization—belonging to the heptazine rings, while one corresponding to a bridging N atom (with formal sp^3^ hybridization) between two heptazine units. The computed hyperfine couplings reproduce fairly well the experimentally observed values (Figure S22), moreover, this structure provides a protonated N site at 0.35 nm, which can act as a compensating charge in the hydrogen mediated redox process. A similar situation holds for Cu^II^, Figure 4d, whereby the shorter M─N bond distance favors a larger spin delocalization over the ligands and consequent larger hyperfine couplings in good agreement with the experimental values (Table 1).

Importantly, while the four coordinated geometry is retained for the Ni^II^/Ni^I^ redox couple, in the case of Cu, reduction from +2 to +1 brings about a significant distortion of the coordination geometry, which may explain the reluctancy in the reoxidation of Cu^I^ (Figure 2) and the suppressed photoredox activity of Cu@CN_x_ as compared to Ni@CN_x_.

In summary, EPR provides detailed insights into the redox chemistry of SACs on CN_x_ and the local coordination environment of their open‐shell electronic states. The isoelectronic Ni^I^@CN_x_ and Cu^II^@CN_x_ species exhibit similar local structure with MN_4_ square planar coordination. The spectroscopic properties are remarkably similar to those reported for Cu^II^ and Ni^I^ porphyrins reinforcing the notion that these materials harbour well defined molecular‐like single atoms. EPR quantification reveals that the number of redox‐active, square–planar coordinated sites is limited and constant irrespective of the overall metal loading. This suggests that CN_x_ provides only a limited number of redox‐active coordination sites. However, Ni@CN_x_ and Cu@CN_x_ exhibit distinct redox behavior. While Ni^II^/Ni^I^ redox cycling is efficient, Cu^II^/Cu^I^ is inhibited. From a ligand‐field perspective, Ni^II^ (d^8^, low‐spin) adopts a square–planar geometry with an empty $d_{x2y2}$ orbital that becomes singly occupied upon reduction to Ni^I^ while preserving a four‐coordinate arrangement. This results in a small inner‐sphere reorganisation energy and enables reversible Ni^II^/Ni^I^ cycling. In contrast, reduction of Cu^II^ (d^9^) to closed‐shell Cu^I^ (d^10^) destabilizes the four‐coordinate field and forces relaxation to a low‐coordinate, quasi‐linear geometry, as confirmed by EXAFS. Returning to a square–planar Cu^II^ N_4_ site therefore requires a substantial structural penalty, suppressing redox reversibility. The catalytic inactivity of Cu@CN_x_ reflects the inability of Cu^I^ to recover the square–planar geometry required for reoxidation.

Notably, XAFS analysis of spent catalysts is fully consistent with DFT simulations.

The convergence of spectroscopic, computational, and catalytic evidence shows that photocatalytic activity in CN_x_‐supported SACs is governed by the redox compatibility of the MN_4_ site, rather than by the nominal oxidation state of the metal alone. The most plausible active sites are edge‐located N_4_ motifs, comprising three sp^2^ nitrogen atoms from the CN_x_ framework and one bridging sp^3^‐coordinating nitrogen, which impose a rigid coordination environment. Within this pocket, only metals that undergo a geometry‐preserving M^II^/M^I^ transition can sustain reversible redox cycling. By probing the intrinsic electronic structure of these sites under well‐defined conditions, EPR/ENDOR and XAS isolate the fundamental redox response of the metal centers, revealing geometric and electronic constraints that persist under catalytic operation despite the complexity of the reaction medium. Redox compatibility within a fixed MN_4_ architecture therefore emerges as the decisive criterion for activity. This insight provides a general guideline for selecting metals whose reduced‐state coordination preferences match the structural constraints of rigid supports and suggests that controlling the density and nature of edge‐type MN_4_ sites in CN_x_ offers a rational route to improve redox‐active single‐atom photocatalysts.

## Conflicts of Interest

The authors declare no conflicts of interest.

## Supporting information




**Supporting File 1**: Additional experimental, spectroscopic, catalytic, and computational data supporting the conclusions of this work are provided in the Supporting Information, together with the corresponding additional references [34, 35, 36, 37, 38, 39, 40, 41, 42, 43, 44, 45, 46, 47, 48, 49, 50, 51, 52, 53].

## Data Availability

The data that support the findings of this study are available from the corresponding author upon reasonable request.

## References

[1] D. K. Böhme and H. Schwarz , “Gas‐Phase Catalysis by Atomic and Cluster Metal Ions: The Ultimate Single‐Site Catalysts,” Angewandte Chemie International Edition 44 (2005): 2336–2354. 15779095 10.1002/anie.200461698

[2] F. Zasada , P. Pietrzyk , M. Radoń , and Z. Sojka , “Mechanistic and Thermodynamic Insights Into Binding and Activation of Small Molecules on Metallozeolites—Relevance for Adsorption and Catalysis,” Chemical Society Reviews 55 (2026): 144–209, 10.1039/D5CS00346F. 41201203

[3] M. Melchionna and P. Fornasiero , “On the Tracks to ‘Smart’ Single‐Atom Catalysts,” Journal of the American Chemical Society 147 (2025): 2275–2290, 10.1021/jacs.4c15803. 39757830 PMC11760184

[4] V. Giulimondi , S. Mitchell , and J. Pérez‐Ramírez , “Challenges and Opportunities in Engineering the Electronic Structure of Single‐Atom Catalysts,” ACS Catalysis 13 (2023): 2981–2997, 10.1021/acscatal.2c05992. 36910873 PMC9990067

[5] G. F. S. R. Rocha , M. A. R. da Silva , A. Rogolino , et al., “Carbon Nitride Based Materials: More Than Just a Support for Single‐Atom Catalysis,” Chemical Society Reviews 52 (2023): 4878–4932, 10.1039/D2CS00806H. 37409655

[6] V. W. Lau and B. V. Lotsch , “A Tour‐Guide Through Carbon Nitride‐Land: Structure‐ and Dimensionality‐Dependent Properties for Photo(Electro)Chemical Energy Conversion and Storage,” Advanced Energy Materials 12 (2022): 2101078, 10.1002/aenm.202101078.

[7] M. Marchi , E. Raciti , S. M. Gali , et al., “Carbon Vacancies Steer the Activity in Dual Ni Carbon Nitride Photocatalysis,” Advancement of Science 10 (2023): 2303781, 10.1002/advs.202303781. PMC10502671 37409444

[8] G. Gentile , M. Marchi , M. Melchionna , P. Fornasiero , M. Prato , and G. Filippini , “Use of Carbon Nitrides as Photoactive Supports in Single‐Atom Heterogeneous Catalysis for Synthetic Purposes,” European Journal of Organic Chemistry 2022 (2022): e202200944, 10.1002/ejoc.202200944.

[9] V. B. Saptal , V. Ruta , M. A. Bajada , and G. Vilé , “Single‐Atom Catalysis in Organic Synthesis,” Angewandte Chemie International Edition 62 (2023): e202219306, 10.1002/anie.202219306. 36918356

[10] H. Schlomberg , J. Kröger , G. Savasci , et al., “Structural Insights Into Poly(Heptazine Imides): A Light‐Storing Carbon Nitride Material for Dark Photocatalysis,” Chemistry of Materials 31 (2019): 7478–7486, 10.1021/acs.chemmater.9b02199. 31582875 PMC6768190

[11] X. Wang , S. Blechert , and M. Antonietti , “Polymeric Graphitic Carbon Nitride for Heterogeneous Photocatalysis,” ACS Catalysis 2 (2012): 1596–1606, 10.1021/cs300240x.

[12] A. Genoux , M. Pauly , C. L. Rooney , et al., “Well‐Defined Iron Sites in Crystalline Carbon Nitride,” Journal of the American Chemical Society 145 (2023): 20739–20744, 10.1021/jacs.3c05417. 37703184

[13] G. Vilé , D. Albani , M. Nachtegaal , et al., “A Stable Single‐Site Palladium Catalyst for Hydrogenations,” Angewandte Chemie International Edition 54 (2015): 11265–11269. 26230282 10.1002/anie.201505073

[14] G. Vilé , N. Allasia , S. Xu , et al., “Unveiling the True Nanostructure of Carbon Nitride‐supported Single‐atom Catalysts,” 2024, Research Square preprint, 10.21203/rs.3.rs-4812493/v1. PMC12160693 39780701

[15] E. I. Solomon and R. G. Hadt , “Recent Advances in Understanding Blue Copper Proteins,” Coordination Chemistry Reviews 255 (2011): 774–789, 10.1016/j.ccr.2010.12.008.

[16] H. B. Gray , B. G. Malmström , and R. J. Williams , “Copper Coordination in Blue Proteins,” Journal of Biological Inorganic Chemistry 5 (2000): 551–559, 10.1007/s007750000146. 11085645

[17] J. D. Cope , H. U. Valle , R. S. Hall , et al., “Tuning the Copper(II)/Copper(I) Redox Potential for More Robust Copper‐Catalyzed C—N Bond Forming Reactions,” European Journal of Inorganic Chemistry 2020 (2020): 1278–1285, 10.1002/ejic.201901269. 33986626 PMC8115207

[18] S. U. Dighe , F. Juliá , A. Luridiana , J. J. Douglas , and D. Leonori , “A Photochemical Dehydrogenative Strategy for Aniline Synthesis,” Nature 584 (2020): 75–81, 10.1038/s41586-020-2539-7. 32760044

[19] P. Ruiz‐Castillo and S. L. Buchwald , “Applications of Palladium‐Catalyzed C–N Cross‐Coupling Reactions,” Chemical Reviews 116 (2016): 12564–12649, 10.1021/acs.chemrev.6b00512. 27689804 PMC5070552

[20] A. Savateev and M. Antonietti , “Ionic Carbon Nitrides in Solar Hydrogen Production and Organic Synthesis: Exciting Chemistry and Economic Advantages,” Chemcatchem 11 (2019): 6166–6176, 10.1002/cctc.201901076.

[21] M. Melchionna and P. Fornasiero , “Updates on the Roadmap for Photocatalysis,” ACS Catalysis 10 (2020): 5493–5501, 10.1021/acscatal.0c01204.

[22] M. J. Strauss , K. X. Liu , M. E. Greaves , et al., “Cu‐Catalyzed Amination of Base‐Sensitive Aryl Bromides and the Chemoselective N‐ and O‐Arylation of Amino Alcohols,” Journal of the American Chemical Society 146 (2024): 18616–18625, 10.1021/jacs.4c05246. 38924516 PMC11375568

[23] S.‐T. Kim , M. J. Strauss , A. Cabré , and S. L. Buchwald , “Room‐Temperature Cu‐Catalyzed Amination of Aryl Bromides Enabled by DFT‐Guided Ligand Design,” Journal of the American Chemical Society 145 (2023): 6966–6975, 10.1021/jacs.3c00500. 36926889 PMC10415864

[24] A. T. Abdulghaffar , H. Zhang , Q. Zhang , et al., “Photoinduced Ullmann‐type Cross‐Coupling Reactions: Mechanistic Insights and Emerging Challenges,” Organic Chemistry Frontiers 12 (2024): 346–367, 10.1039/D4QO01814A.

[25] B. T. Phelan , M. W. Mara , and L. X. Chen , “Excited‐State Structural Dynamics of Nickel Complexes Probed by Optical and X‐Ray Transient Absorption Spectroscopies: Insights and Implications,” Chemical Communications 57 (2021): 11904–11921, 10.1039/D1CC03875C. 34695174

[26] X. Zhang , H. Su , P. Cui , et al., “Developing Ni Single‐Atom Sites in Carbon Nitride for Efficient Photocatalytic H2O2 Production,” Nature Communications 14 (2023): 7115, 10.1038/s41467-023-42887-y. PMC10628073 37932292

[27] J. T. Moore , N. E. Smith , and C. C. Lu , “Structure and Dynamic NMR Behavior of Rhodium Complexes Supported by Lewis Acidic Group 13 Metallatranes,” Dalton Transactions 46 (2017): 5689–5701, 10.1039/C6DT04769F. 28239706

[28] P. Pietrzyk , T. Mazur , K. Podolska‐Serafin , M. Chiesa , and Z. Sojka , “Intimate Binding Mechanism and Structure of Trigonal Nickel(I) Monocarbonyl Adducts in ZSM‐5 Zeolite—Spectroscopic Continuous Wave EPR, HYSCORE, and IR Studies Refined With DFT Quantification of Disentangled Electron and Spin Density Redistributions Along σ and π Channels,” Journal of the American Chemical Society 135 (2013): 15467–15478. 24044734 10.1021/ja405874t

[29] T. Jia , D. Meng , R. Duan , et al., “Single‐Atom Nickel on Carbon Nitride Photocatalyst Achieves Semihydrogenation of Alkynes With Water Protons via Monovalent Nickel,” Angewandte Chemie International Edition 62 (2023): e202216511, 10.1002/anie.202216511. 36625466

[30] J. Telser , Y.‐C. Fann , M. W. Renner , et al., “Investigation by EPR and ENDOR Spectroscopy of the Nickel(I) Form of Cofactor F4301 of Methanobacterium Thermoautotrophicum and of Nickel(I) Octaethylisobacteriochlorin,” Journal of the American Chemical Society 119 (1997): 733–743, 10.1021/ja9625337.

[31] C. Finazzo , J. Harmer , B. Jaun , et al., “Characterization of the MCRred2 Form of Methyl‐Coenzyme M Reductase: A Pulse EPR and ENDOR Study,” Journal of Biological Inorganic Chemistry 8 (2003): 586–593, 10.1007/s00775-003-0450-y. 12624730

[32] F. Santanni , A. Albino , M. Atzori , et al., “Probing Vibrational Symmetry Effects and Nuclear Spin Economy Principles in Molecular Spin Qubits,” Inorganic Chemistry 60 (2021): 140–151, 10.1021/acs.inorgchem.0c02573. 33305944 PMC7872321

[33] T. G. Brown and B. M. Hoffman , “14N, 1H, and Metal ENDOR of Single Crystal Ag(II)(TPP) and Cu(II)(TPP),” Molecular Physics 39 (1980): 1073–1109, 10.1080/00268978000100911.

[34] B. Ravel and M. Newville , “ATHENA, ARTEMIS, HEPHAESTUS: Data Analysis for X‐Ray Absorption Spectroscopy Using IFEFFIT,” Journal of Synchrotron Radiation 12 (2005): 537–541, 10.1107/S0909049505012719. 15968136

[35] J. J. Rehr , R. C. Albers , and S. I. Zabinsky , “High‐Order Multiple‐Scattering Calculations of X‐Ray‐Absorption Fine Structure,” Physical Review Letter 69 (1992): 3397–3400, 10.1103/PhysRevLett.69.3397. 10046808

[36] T. Kyômen , R. Yamazaki , and M. Itoh , “Valence and Spin state of Co and Ni Ions and Their Relation to Metallicity and Ferromagnetism in LaNi0.5Co0.5O3,” Physical Review B 68 (2003): 104416.

[37] J. Rabeah , J. Radnik , V. Briois , et al., “Tracing Active Sites in Supported Ni Catalysts During Butene Oligomerization by Operando Spectroscopy Under Pressure,” ACS Catalysis 6 (2016): 8224–8228, 10.1021/acscatal.6b02331.

[38] N. Rossetti , A. Ugolotti , C. Cometto , et al., “Insights Into the Active Nickel Centers Embedded in Graphitic Carbon Nitride for the Oxygen Evolution Reaction,” Journal of Materials Chemistry A 12 (2024): 6652–6662, 10.1039/D3TA07389K.

[39] F. W. Lytle , R. B. Greegor , and A. J. Panson , “Discussion of X‐Ray‐Absorption Near‐Edge Structure: Application to Cu in the High‐Tc Superconductors La1.8Sr0.2CuO4 and YBa2Cu3O7,” Physical Review B 37 (1988): 1550–1562, 10.1103/PhysRevB.37.1550. 9944673

[40] C. Maurizio , F. d'Acapito , M. Benfatto , S. Mobilio , E. Cattaruzza , and F. Gonella , “Local Coordination Geometry Around Cu and Cu Ions in Silicate Glasses: An X‐Ray Absorption Near Edge Structure Investigation,” European Physical Journal B: Condensed Matter and Complex Systems 14 (2000): 211–216, 10.1007/s100510050122.

[41] A. Manceau and A. Matynia , “The Nature of Cu Bonding to Natural Organic Matter,” Geochimica Et Cosmochimica Acta 74 (2010): 2556–2580, 10.1016/j.gca.2010.01.027.

[42] X. Dai , Y. Han , H. Jiao , F. Shi , J. Rabeah , and A. Brückner , “Aerobic Oxidative Synthesis of Formamides From Amines and Bioderived Formyl Surrogates,” Angewandte Chemie International Edition 63 (2024): e202402241, 10.1002/anie.202402241. 38567831

[43] C. Cometto , A. Ugolotti , E. Grazietti , et al., “Copper Single‐Atoms Embedded in 2D Graphitic Carbon Nitride for the CO_2_ Reduction,” NPJ 2D Materials and Applications 5 (2021): 63, 10.1038/s41699-021-00243-y.

[44] P. Schosseler , T. H. Wacker , and A. Schweiger , “Pulsed ELDOR Detected NMR,” Chemical Physics Letters 224 (1994): 319–324, 10.1016/0009-2614(94)00548-6.

[45] N. Cox , A. Nalepa , W. Lubitz , and A. Savitsky , “ELDOR‐detected NMR: A General and Robust Method for Electron‐Nuclear Hyperfine Spectroscopy?,” Journal of Magnetic Resonance 280 (2017): 63–78, 10.1016/j.jmr.2017.04.006. 28579103

[46] P. Höfer , A. Grupp , H. Nebenführ , and M. Mehring , “Hyperfine Sublevel Correlation (hyscore) Spectroscopy: A 2D ESR Investigation of the Squaric Acid Radical,” Chemical Physics Letters 132 (1986): 279–282, 10.1016/0009-2614(86)80124-5.

[47] S. Stoll and A. Schweiger , “EasySpin, a Comprehensive Software Package for Spectral Simulation and Analysis in EPR,” Journal of Magnetic Resonance 178 (2006): 42–55, 10.1016/j.jmr.2005.08.013. 16188474

[48] J. P. Perdew , K. Burke , and M. Ernzerhof , “Generalized Gradient Approximation Made Simple,” Physical Review Letter 77 (1996): 3865–3868, 10.1103/PhysRevLett.77.3865. 10062328

[49] S. Grimme , J. Antony , S. Ehrlich , and H. Krieg , “A Consistent and Accurate *Ab Initio* Parametrization of Density Functional Dispersion Correction (DFT‐D) for the 94 Elements H‐Pu,” Journal of Chemical Physics 132 (2010): 154104, 10.1063/1.3382344. 20423165

[50] F. Weigend and R. Ahlrichs , “Balanced Basis Sets of Split Valence, Triple Zeta Valence and Quadruple Zeta Valence Quality for H to Rn: Design and Assessment of Accuracy,” Physical Chemistry Chemical Physics 7 (2005): 3297, 10.1039/b508541a. 16240044

[51] S. Sinnecker , L. D. Slep , E. Bill , and F. Neese , “Performance of Nonrelativistic and Quasi‐Relativistic Hybrid DFT for the Prediction of Electric and Magnetic Hyperfine Parameters in 57Fe Mössbauer Spectra,” Inorganic Chemistry 44 (2005): 2245–2254, 10.1021/ic048609e. 15792459

[52] F. Neese , “Software Update: The ORCA Program System—Version 5.0,” WIREs Computational Molecular Science 12 (2022): e1606, 10.1002/wcms.1606.

[53] N. Allasia , S. Xu , S. F. Jafri , et al., “Resolving the Nanostructure of Carbon Nitride‐Supported Single‐Atom Catalysts,” Small 21 (2025): 2408286, 10.1002/smll.202408286. 39780701 PMC12160693

