# Supplementary material for: On the Structure and Redox Behavior of Ni and Cu Single Atoms Supported on Carbon Nitride
Source: Angew Chem Int Ed Engl. 2026 Apr 2;65(19):e2299555. doi: 10.1002/anie.2299555 (PMC13134600; doi:10.1002/anie.2299555)
Supplement: Supplementary file 1 — Supporting File 1: Additional experimental, spectroscopic, catalytic, and computational data supporting the conclusions of this work are provided in the Supporting Information, together with the corresponding additional references [34, 35, 36, 37, 38, 39, 40, 41, 42, 43, 44, 45, 46, 47, 48, 49, 50, 51, 52, 53]. [file ANIE-65-e2299555-s001.docx]

Supporting Information

On the Structure and Redox Behaviour of Ni and Cu Single Atoms Supported on Carbon Nitride

Giovanni Colonnello^[a,b]+^, Ksenija Maver^[a]+^, Arianna Actis^[a]^, Gaia Grando^[c]^, Giacomo Filippini^[c]^, Tiziano Montini^[c]^, Michele Melchionna^[c,d]^, Paolo Fornasiero^[c]^, Lucia Nasi^[e]^, Iztok Arčon^[f]^, Lorenzo Donà^[a]^, Bartolomeo Civalleri^[a]^, Enrico Salvadori^[a]^, Mario Chiesa*^[a]^.

^+^ These authors made equal contribution

[a] Mr. G. Colonnello, Dr. K. Maver, Dr. A. Actis, Dr. L. Donà, Prof. B. Civalleri, Prof. E. Salvadori, Prof. M Chiesa
Department of Chemistry University of Torino Via Giuria 7, 10125, Torino, Italy. E-mail: [mario.chiesa@unito.it](mailto:mario.chiesa@unito.it)

[b] Mr. G. Colonnello DII – Department of Industrial Engineering, University of Padova, via Marzolo 9, 35131 Padova, Italy

[c] Mrs. G. Grando, Dr. G. Filippini, Prof. M. Melchionna, Prof. Paolo Fornasiero
Department of Chemical and Pharmaceutical Sciences, Center for Energy, University of Trieste, INSTM Trieste Research Unit, Via Licio Giorgieri 1, 34127 Trieste, Italy

[d] Prof. Michele Melchionna Nanotechnology Centre, Centre for Energy and Environmental Technologies, VŠB–Technical University of Ostrava, 17. listopadu 2172/15, 708 00 Ostrava-Poruba, Czech Republic

[e] Dr. Lucia Nasi Institute of Materials for Electronics and Magnetism, National Research Council (IMEM-CNR),

Parco Area delle Scienze 37/A, 43124 Parma, Italy

[f] Prof. Iztok Arčon University of Nova Gorica Vipavska 13, SI-5000 Nova Gorica, Slovenia and Jožef Stefan Institute Jamova 39, SI-1000 Ljubljana, Slovenia

Table of Contents

S[1. Experimental methods and materials characterization data. 3](#_Toc155883049)

S[1.1. Synthesis of Ni and Cu/CN catalyststs. 3](#_Toc155883050)

S[1.2 XAS experiments. 3](#_Toc155883051)

S[1.3 EPR experiments. 5](#_Toc155883052)

# S1.4 Physisorption Measurements 5

# S1.5 Inductively Coupled Plasma-Optical Emission Spectroscopy ICP-OES 5

S[1.6 Electron Microscopy Characterization. 6](#_Toc155883053)

S[2. Catalytic test reaction. 6](#_Toc155883054)

S[3. DFT calculation methods. 7](#_Toc155883055)

S[4. Figures S1-S31. 8](#_Toc155883056)

S[5. Tables S1-S5.](#_Toc155883056) 39

S6. [References 43](#_Toc155883057)

S1. Experimental methods and materials characterization data

S1.1 Synthesis of Ni and Cu/CN catalysts.

**Materials and methods**: Commercial precursors and solvents were purchased from Sigma-Aldrich, Fluka, Alfa Aesar, Fluorochem, VWR and used as received. The microwave synthesis was performed on a CEM Discover-SP.

**Synthesis**: The materials were obtained in a three steps synthesis. i) graphitic carbon nitride (CN_x_) was synthetized from melamine, then ii) the material was modified with microwave treatment (*mw*-CN_x_), and iii) the material was impregnated with the metals. The procedure of the first two steps is the one reported in previous works,^[1]^ and here summarized.

CN_x_: 10 g of melamine were heated in muffle furnace up to 550 °C for 300 min in a covered alumina crucible with a ramping time of 5 °C min^−1^. The final product was milled in a mortar to obtain a fine and uniform powder.

*mw*-CN_x_: 200 mg of CN_x_ were heated at 190 °C for 45 min by microwave irradiation. The sample was collected by filtration and dried at 80 °C overnight.

Ni@CN_x_ and Cu@CN_x_: into a 500 ml round bottomed flask, equipped with a stirring bar were added: 0.5 g of *mw*-CN_x_, 40 mmol of methyl 4-bromobenzoate (8.6 g), different amount of the metal salts (Ni(NO_3_)_2_ x 6H_2_O or Cu(NO_3_)_2_ x 3H_2_O) were added to have different single atom (SA) metal loadings, and 200 ml of DMF. The resulting mixture was degassed bubbling argon for 20 min. After that 120 mmol (10 ml) of pyrrolidine, were added. The mixture was left under stirring for 24h, inert atmosphere was maintained with an Ar filled ballon. The solid was then recovered by centrifugation (15 min, 5000 rpm), washed two times with DMF (2 x 20 ml), five times with bidistilled water (5 x 20 ml) and one time with ethanol (20 ml). The washing was performed by suspending the material in ultrasound bath for 10 min and then redeposit with centrifuge (10 min, 5000/5500 rpm). The clean product was dried overnight at 60 °C, and then milled in an agate mortar. A schematic representation of the metal impregnation procedure can be seen in Figure S1.

S1.2. XAS experiments.

X-ray absorption spectroscopy (XAS) was conducted on the P65 beamline at PETRA III (Desy, Hamburg). A Si(111) double crystal monochromator was used with an energy resolution of about 1 eV at 8 keV. The beam size on the sample was 0.3 mm x 1 mm. Higher-order harmonics were effectively eliminated by a flat mirror. Up to 5 repetitions of XAS spectra were measured in continuous detection mode in short 3-minute scans of the catalyst samples. The intensity of the monochromatic X-ray beam was measured with the ionization detectors (I1, I2 and I3) filled with 1020 mbar of suitable absorption gas mixtures to absorb a total of 16 % (10 % Ar, 90 % N2), 54 % (15 % Ar, 85 % N2) and 90 % (Kr), respectively. The absorption spectra were measured in the energy region from −150 eV to +1000 eV relative to the Ni and Cu K-edge (8333 and 8979 eV, respectively). The exact energy calibration was established with absorption measurement on a 5-micron thick Ni and Cu metal foil, respectively. The absolute energy reproducibility of the measured spectra was ±0.05 eV. The as-prepared and reduced Ni@CN_x_ and Cu@CN_x_ sample catalysts in powder forms were inserted in a thin quartz EPR tubes and sealed in vacuum or H_2_ atmosphere. In this way, the XAS measurements were coordinated with the EPR experiments. The reference compounds (crystalline NiO, crystalline Cu^I^ oxide (Cu_2_O) and crystalline Cu^II^ oxide (CuO)) were homogeneously mixed with 50 mg of boron nitride and prepared in the form of homogeneous pellets with an optimal total absorption thickness of about 2 above the Ni and Cu K-edge.

The analysis of XAS spectra was performed with the DEMETER (IFEFFIT) program package^[2]^ with the FEFF8 program code^[3]^ for the ab initio calculation of photoelectron scattering paths.

**S1.2.1 Ni-K edge**

**XANES region** Ni K-edge X-ray absorption near edge structure (XANES) analysis was used to monitor the valence state and local symmetry around Ni cations grafted onto the carbon nitride (CN_x_) support. The energy position of the Ni K-edge is correlated with the valence state of the Ni cations in the sample and is shifted to higher energies by about 2 eV per valence state.^[4]^

The XANES spectra of the as-prepared and reduced catalyst are shown together with the XANES spectra of the reference compounds (Figure 1 main text): Ni metal foil and crystalline NiO as a reference for Ni^0^ and Ni^II^ cations respectively. The Ni K-edge energy position of the of the as-prepared catalysts is at the position of the reference NiO, which means that the Ni cations are predominantly present in divalent form. The XANES spectrum of the H_2_-reduced catalyst is between metal foil (Ni^0^) and crystalline NiO (reference for Ni^II^), which suggests that Ni^I^ are the dominant Ni species.^[5]^

Moreover, the linear combination fitting (LCF) analysis,^[2]^ not shown here, with the reference spectra of metal foil and crystalline NiO could not describe the catalyst edge profiles, which means that the H_2_-reduced Ni@CN_x_ sample does not contain a mixture of metallic Ni^0^ and Ni^II^ oxide (NiO) particles. From this we can conclude that the Ni cations in the as-prepared catalyst were successfully reduced after H_2_-treatment.

**EXAFS analysis.** Ni K-edge extended X-ray absorption fine structure (EXAFS) analysis was used to observe the changes in the local structure around the nickel in the as-prepared sample and after H_2_ reduction. Since the spectra were measured in a thin capillary, the EXAFS region can only be examined in the *k*-region up to 9 Å^−1^ and 8 Å^−1^ case of as-prepared and H_2_-reduced sample, respectively.

The FEFF model for the as-prepared sample is based on the crystal structure of NiO with space group *Fm3m*, in which Ni is coordinated with 6 oxygen atoms at 2.08 Å, 12 Ni atoms at 2.95 Å. The FEFF model includes two single scattering paths up to 3.2 Å. According to the EPR results (see results below), we introduced N atoms in the first shell instead of O, although N and O cannot be distinguished in the EXAFS analysis due to the very similar backscattering factor. We used 8 variables: coordination shell distances (Δ*r*) and Debye-Waller factors (*σ^2^*) of both single scattering paths, the shift of the energy origin of the photoelectron (Δ*E*_o_) and the amplitude reduction factor (*S*_0_^2^) were common to the scattering paths. The average coordination numbers (*N*) of the coordination shells were allowed to vary and the amplitude reduction factor (*S*_0_^2^) was set to 0.73. The model was confirmed using the EXAFS spectrum measured on the reference crystalline NiO (Figure S2a). For the as-prepared sample (Figure S2b) a good EXAFS fit was obtained in the *k*-range of 3–9 Å^−1^ and in the *R*-range of 1.2–3.2 Å. The best fitted structural parameters are listed in Table S3.

The results of the EXAFS analysis show that in the as-prepared catalysts (Table S3, Figure S2b) the Ni cations are coordinated with nitrogen atoms in the first shell with coordination numbers lower and the Debye-Waller factors higher than for nanocrystalline NiO. (Table S3).

The FEFF model for the reduced catalysts is based on the EPR and DFT results which indicate a structure with square-planar configuration, where Ni is coordinated to 4 nitrogen atoms at distances of the order of 2 Å. We included only one scattering path for the first coordination shell with four nitrogen atoms from the FEFF model because the signal-to-noise ratio was too weak to model more distant neighbours. Four variables were allowed to vary: the coordination number (*N*) of the nitrogen shell, the coordination shell distance (*Δr*), the Debye-Waller factor (*σ^2^*) and the shift of the energy origin of the photoelectron (Δ*E*_o_). The amplitude reduction factor (*S_0_^2^*) was set to 0.73. The fitting was performed in the *k*-range of 3–8 Å^−1^ and in the *R*-range of 1.2–2.2 Å (Figure S2c). The best fitted structural parameters are listed in Table S3.

The results of the EXAFS analysis (Table S3, Figure S2c) show that the Ni cations in the H_2_-reduced catalysts are coordinated with four nitrogen neighbours. The distances are in line with the computed models (main text) and comparable to those found in the literature for similar SAC materials.^[6,7]^

**S1.2.2 Cu-K edge**

**XANES region**. The Cu K-edge XANES analysis was used to reveal changes in valence of Cu species and local symmetry of Cu cations in in the as-prepared catalyst and after activation in H_2_ atmosphere (heating from room temperature to 225°C and 300°C in H_2_ atmosphere).

The normalized Cu K-edge XANES spectra of the samples are shown in Figure 1 of the main text together with the spectra of Cu reference compounds: Cu metal foil, crystalline Cu_2_O and crystalline CuO. The valence state of Cu cation can be determined from the energy position of the Cu K-edge. The changes in energy (about 4 eV) of the Cu K-edge is observed between Cu^I^ and Cu^II^ cations, while a smaller edge shift of about 1 eV is exhibited between metallic Cu^0^ and Cu^I^ cation.^[8]^ The energy position of Cu K-edge in the as-prepared and H_2_-treated samples are close to the energy position of Cu^I^ reference compounds, i.e. Cu_2_O (Figure S3), or slightly above. From this we can conclude that Cu cations in the as prepared catalyst are present in both Cu^I^ and Cu^II^ valence states.

In the Cu@CN_x_ catalysts, the intensity of a distinct Cu 1s to 4p pre-edge absorption feature at an energy of 8982.5 eV increases with thermal treatment in H_2_ atmosphere (Figure S3). A well-defined absorption feature also occurs in the reference crystalline Cu_2_O at 8982 eV.^[8,9]^ In the case of Cu^II^ compounds, the 1s to4p transition appears as a pre-edge shoulder in the energy range from 8985 eV to 8989 eV and its intensity increases from distorted octahedral to square planar Cu coordination.^[8,10]^ However, the linear combination fitting (not shown here) with the reference spectra of crystalline Cu_2_O and CuO could not describe the catalyst edge profiles because the absorption feature exceeds the intensity of both reference compounds. This means that the symmetry of the Cu cations in the catalyst differs from these reference species. The EXAFS analysis (below) revealed that the peak can be attributed to the formation of 2N linearly coordinated Cu^I^ species upon reduction.

**EXAFS analysis.** Cu K-edge EXAFS analysis was used to determine the average local structure around the copper cations in Cu@CN_x_ catalysts. We investigated the changes in the local structure before (the as-prepared sample) and after H_2_-reduction at 225 °C and 300 °C.

In the Fourier transform EXAFS spectra (Figure S4), the contributions of photoelectron scattering at the nearest neighbor shells around the Cu atoms are observed in the R region up to about 2.8 Å. According to EPR results, we attribute the strong peak in the R region between 1.2 Å and 2.0 Å to photoelectron backscattering from the nearest nitrogen neighbors around Cu. Weaker peaks in the R range between 2.0 Å and 2.8 Å represent the contributions from more distant coordination shells.

The FEFF model for the Cu@CN_x_ samples is based on the EPR spectrum and related DFT models, indicating square planar coordination of Cu^II^ to four N ligands and C neighbors in more distant coordination shells, at distances characteristic of copper grafted to g-C_3_N_4_.^[11,12]^

The FEFF model includes two single scattering paths up to 2.8 Å. We used 6 variables: coordination shell distances (Δ*r*) and Debye-Waller factors (*σ^2^*) of both single scattering paths, the shift of the energy origin of the photoelectron (Δ*E*_o_) and the amplitude reduction factor (*S*_0_^2^) were common to the scattering paths. The average coordination numbers (*N*) of the coordination shells were allowed to vary and the amplitude reduction factor (*S*_0_^2^) was set to 0.85. The best fit of the FT EXAFS spectra are shown in Figure S4 a-c and the list of the corresponding best fit parameters can be found in Table S4.

The results of the EXAFS analysis show that in the coordination number in the first shell goes from 4 nitrogen atoms in the case of the as-prepared sample to 2 nitrogen atoms in the case of the sample reduced in H_2_ at 300 °C. For the sample reduced at 250 °C, the coordination number is 2.5, indicating a mixture of 4 and 2 coordinated Cu. These results very well support the EPR and XANES findings. According to EPR, Cu^II^ is observed only in the as-prepared and catalysts treated up to 225 °C in H_2_ atmosphere, which indicate that Cu^II^ are present in 4-fold coordination of N atoms. Cu^I^ becomes EPR-silent when the samples are reduced in H_2_ at 300 °C, which indicates that all available Cu are reduced to Cu^I^ attaining two-fold coordination. The XANES results also showed that the Cu cations in the catalysts are in a mixture of mono- and bi-valent state, in agreement with the EPR results.

S1.3. EPR experiments.

For CW EPR experiments, EPR cells that can be connected to conventional high-vacuum apparatus (residual pressure <10^–4^ mbar) were employed. For reduction and oxidation 20 mbars of respectively H_2_ and O_2_ were dosed in the EPR cell. The cells were heated with a steady 5°C/min ramp from room temperature to 300°C and measured every 50°C.

X-band (microwave frequency 9.45 GHz) CW EPR measurements were performed on a Bruker EMX spectrometer equipped with an ER 4122 SHQ cylindrical cavity. A modulation frequency of 100 kHz, a modulation amplitude of 0.5 mT, and a microwave power of 1 mW (for spectra at 77 K) and of 10 mW (for spectra at room temperature) were used.

Q-band (microwave frequency 33.7 GHz) CW EPR experiments were performed on a Bruker Elexsys E580 spectrometer equipped with an EN 5107D2 Bruker resonator, an Oxford Instruments CF935 helium-flow cryostat and an ITC503 temperature controller. Q-band electron-spin-echo (ESE) detected EPR spectra were recorded with the pulse sequence π/2–τ–π–τ–echo. Pulse lengths t_π/2_ = 16 ns and t_π_ = 32 ns, a τ value of 200 ns was used.

EDNMR experiments were obtained with the pulse sequence: HTA – *T* – π/2 – τ – π – τ – echo,^[13]^ with *t*_HTA_ = 9000 ns, *T* = 1000 ns, *t*_π/2_ = 400 ns, and τ = 600 ns. A Gaussian-shaped HTA pulse, as generated by a Bruker SpinJet-AWG, was used. The integration window was set to 800 ns centered around the maximum of the spin echo. The central hole at Δν = 0 was removed in the EDNMR spectra by subtracting a fitted Lorentzian line shape. Simulation of the EDNMR spectra was performed using a simulation algorithm based on the work by Cox et al.^[14]^

Q-band ENDOR spectra were recorded with both Mims and Davies pulse sequences. Davies ENDOR used the pulse sequence π–T–π/2–τ–π–τ–echo with t_π_ = 32 ns and t_π/2_ = 16 ns, t_πRF_ = 14 μs, T = 16 μs and τ = 200 ns. Q-band Mims ENDOR employed the pulse sequence π/2–τ–π/2–π_RF_–π/2–τ–echo with t_π/2_ = 16 ns, τ = 150 ns, t_πRF_ = 14 μs and additional waiting times of 1 μs were used before and after the π_RF_ pulse. Mims ENDOR spectra were recorded in a 15 MHz window centered at the 1 H Larmor frequency, with a 0.043 MHz spectral resolution.

HYSCORE spectroscopy Q-band Hyperfine Sublevel Correlation (HYSCORE) spectroscopy^[15]^ experiments were carried out with the standard pulse sequence π/2–τ–π/2–t1–π–t2–π/2–τ–echo, applying an eight-step phase cycle for eliminating unwanted echoes. Microwave pulse lengths t_π/2_ = 16 ns, t_π_ = 32 ns, and a shot repetition rate of 1.0 kHz were used. The t1 and t2 time intervals were incremented in steps of 8 ns, starting from 150 ns, giving a data matrix of 256 x 256 points. The time traces of the HYSCORE spectra were baseline corrected with a third-order polynomial, apodised with a Hamming window and zero filled. After two-dimensional Fourier transformation, the absolute value spectra were calculated. The τ value used for each measurement are specified in the figure captions.

All spectra were simulated using the EasySpin^[16]^ package running in Matlab.

# S1.4 Physisorption Measurements

# N_2_ Adsorption/desorption isotherms (Figure S5) were obtained using 3FLEX Surface Characterization from Micrometrics. Before analysis, the materials were degassed in vacuum with turbo pump, to ensure the complete removal of adsorbed species. The isotherms were collected at liquid nitrogen temperature (LNT), using N2 as adsorptive gas. The surface areas of the materials were then determined using Brunauer-Emmett-Teller (BET) model.

# S1.5 Inductively Coupled Plasma-Optical Emission Spectroscopy ICP-OES

Inductively Coupled Plasma-Optical Emission Spectroscopy (ICP-OES) analyses were performed using an Optima 8000 Spectrometer equipped with a S10 autosampler (PerkinElmer, USA). To perform the analysis 10 or 100 mg of the samples were calcinated at 900 °C in air to decompose all the CN matrix, then the residual powder was treated in aqua regia solution (1 mL) overnight to dissolve the metal powder, afterwards, the same solution was diluted 1 : 10 in bidistilled water. The total metal concentration was quantified after instrument calibration, performed by diluting the multistandard solution for ICP analysis (Periodic Table MIX1, Merck). All standards (range: 0–10.0 mg/L) were prepared by acidifying to 10 % with aqua regia to compensate for the matrix effect. The limits of detection at the operative wavelengths (Cu: 327.393 nm; Ni: 231.604 nm) were 0.02 mg/L for both elements. The precision of the measurements expressed as repeatability (as RSD%) for the analysis was always less than 5 %.

S1.6. Electron Microscopy Characterization.

The presence of the metal was revealed in both Ni@CN_x_ and Cu@CN_x_ using either Electron Energy Loss Spectroscopy (EELS) than energy dispersive X-ray spectroscopy (EDS). In the EDS maps (Figure S6) the metals appear uniformly distributed on the support, while high resolution TEM (HRTEM) and scanning transmission electron microscopy (STEM) do not reveal any metal crystalline aggregates. These results are consistent with a single-atom dispersion. However, in the STEM-HAADF images no "white dots", typically assigned to the isolated metal atoms, are distinctly observed, this being of particular challenge for metal atoms with small Z contrast with respect to support.

S1.7. X-ray powder Diffraction

XRD was performed on a Philips X’Pert diffractometer using a monochromatized Cu Kα (λ = 0.154 nm) x-ray source, with a Bragg-Bentano geometry and a step of 0.017° in the range of 5° < 2θ < 40°.

S2. Catalytic Test Reactions.

**Materials:** Commercial reagents and solvents were purchased from Sigma-Aldrich, Fluka, Alfa Aesar, Fluorochem, VWR and used as received, without further purification, unless otherwise stated. The photochemical reactions were set up under an argon atmosphere in Schlenk tubes. Chromatographic purification of products was accomplished using flash chromatography on silica gel (SiO_2_, 0.04–0.063 mm, 60 Å) purchased from Machery-Nagel, with the indicated solvent system according to the standard techniques or using a Biotage Isolera automated flash chromatography system with cartridges packed with SiO_2_ (0.04-0.063 mm, 60 Å). Thin-layer chromatography (TLC) analysis was performed on precoated Merck TLC plates (silica gel 60 GF254, 0.25 mm). Visualization of the developed chromatography was performed by checking UV absorbance (254 nm). Organic solutions were concentrated under reduced pressure on a Büchi rotary evaporator. The irradiation was performed using a Kessil lamp PR160L-456 (50 W) purchased from Kessil webpage: https://kessil.com/products/science_PR160L.php as light source at 456 nm. Synthesis grade and anhydrous solvents were used as purchased. NMR spectra were recorded on a Bruker 400 Avance III HD equipped with a BBI-z grad probe head 5mm (^1^H: 400 MHz, ^13^C: 100.5 MHz). The chemical shifts (*δ*) for ^1^H and ^13^C are given in ppm relative to residual signals of the solvents (CHCl_3_ @ 7.26 ppm for ^1^H NMR, and @ 77.16 ppm for ^13^C NMR). Coupling constants are given in hertz. The following abbreviations are used to indicate the multiplicity: s, singlet; d, doublet; t, triplet; m, multiplet. NMR yields were calculated by using 1,3,5-trimethoxybenzene as internal standard. High-resolution mass spectra (HRMS) were obtained using a Bruker micrOTOF-Q (ESI-TOF)

**General procedure of the reaction**: For the C-N coupling reaction a 10 mL Schlenk tube was charged with the appropriate aryl halide (methyl 4-bromobenzoato or methyl 4-Iodobenzoate) **1a-b** (0.1 mmol, 1 equiv.), pyrrolidine **2a** (0.3 mmol, 3 equiv.), CN_x_ (2.5 mg/mL), metal(II) salts (Ni(NO_3_)_2_ x 6H_2_O or Cu(NO_3_)_2_ x 3H_2_O; 5% mol) and DMF (2M corresponding to 0.5 ml). For the C-O and C-S coupling reactions a 10 mL Schlenk tube was charged with the appropriate aryl halide **1a** (0.1 mmol, 1 equiv.), the corresponding alcohol **2b** or thiol **2c** (0.4 mmol, 4 equiv.), the g-CN photocatalyst (2.5 mg/mL), (Ni(NO_3_)_2_ x 6H_2_O or Cu(NO_3_)_2_ x 3H_2_O; 5% mol), diisopropyl amine (0.5 mmol, 5 equiv.), 4,4’-di-tertbutyl-2,2’-dipyridyl (dtbbpy, 0.01 mmol, 10 mol%), and DMF (0.2 M). The reaction mixture was thoroughly degassed via 4 cycles of freeze-pump-thaw, and the vessel was refilled with argon, and irradiated with blue light (λ = 456 nm, Kessil lamp). The temperature was kept at around 40°C by using a fan (see reaction set up in Figure S8). Stirring was maintained for 8 or 24 hours, then the irradiation was stopped. The reaction mixture was diluted with aqueous LiCl (5% wt/v) and extracted with ethyl acetate. The combined organic layers were dried over sodium sulfate and the solvent were removed in vacuo. The residue was purified by column chromatography (eluent: cyclohexane/ethyl acetate (95 : 5) %) to give the corresponding aniline products **3a-c**.

**Products characterization data**

**Methyl 4-(pyrrolidin-1-yl)benzoate (3a)**


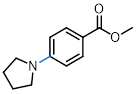
**3a** was synthesized according to the general procedure for C-N coupling from methyl 4- bromobenzoate (21.5 mg, 0.1 mmol, 1 equiv.) or methyl 4-iodobenzoate (22.9 mg, 0.1 mmol, 1 equiv.) and pyrrolidine (25.0 µl, 0.3 mmol, 3 equiv.). The characterization is in accordance with the literature.^[17]^ ^1^H-NMR (400 MHz, CDCl_3_) *δ* 7.90 (m, *J* = 9.0 Hz, 2H), 6.50 (m, *J* = 9.0 Hz, 2H), 3.85 (s, 3H), 3.36 – 3.33 (m, 4H), 2.04 – 2.01 (m, 4H); ^13^C-NMR (101 MHz, CDCl_3_) *δ* 167.73 (s), 150.85 (s), 131.53 (s), 116.76 (s), 111.03 (s), 51.59 (s), 47.89 (s), 25.58 (s); HRMS (ESI, positive mode) calculated for C_12_H_15_NO_2_ [M+Na]^+^: 228.0994, found: 228.0995.

**4-Benzyloxy-benzoic acid methyl ester (3b)**

**
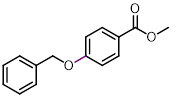
3b** was synthesized according to the general procedure for C-O coupling from methyl 4-iodobenzoate (22.9 mg, 0.1 mmol, 1 equiv.) and benzyl alcohol (42 µl, 0.4 mmol, 4 equiv.). The characterization is in accordance with the literature.^[17]^ ^1^H-NMR (400 MHz, CDCl_3_) δ 8.00 (d, J = 8.8 Hz, 2H), 7.45 – 7.32 (m, 5H), 7.00 (d, J = 8.8 Hz, 2H), 5.12 (s, 2H), 3.89 (s, 3H) ); ^13^C-NMR (101 MHz, CDCl3) δ 166.95 (s), 162.62 (s), 136.39 (s), 131.74 (s), 128.81 (s), 128.34 (s), 127.62 (s), 122.98 (s), 114.59 (s), 70.23 (s), 52.00 (s). HRMS (ESI, positive mode) calculated for C_13_H_14_O_3_ [M+Na]^+^ : 265.0835, found: 265.0837.

**Methyl 4-(octylthio)benzoate (3c)**

**
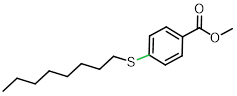
3c** was synthesized according to the general procedure for C-S coupling from methyl 4-iodobenzoate (22.9 mg, 0.1 mmol, 1 equiv.) and 1-octanethiol (87 µl, 0.4 mmol, 4 equiv.). Reaction time: 24 hours. ^1^H NMR (600 MHz, CDCl_3_) δ 7.91 (d, 2H), 7.29 (d, *J* = 4.2 Hz, 2H), 3.90 (s, 3H), 2.97 (t, *J* = 7.4 Hz, 2H), 1.74 – 1.63 (m, 2H), 1.48 – 1.40 (m, 2H), 1.34 – 1.24 (m, 8H), 0.87 (d, *J* = 7.1 Hz, 3H). ^13^C NMR (151 MHz, CDCl3) δ 165.84 (s), 143.51(s), 128.87 (s), 125.50 (s), 125.26 (s), 51.00 (s), 31.06 (s), 30.76 (s), 28.12 (s), 28.09 (s), 27.88 (s), 27.73 (s), 21.61 (s), 13.06 (s). HRMS (ESI, positive mode) calculated for C_16_H_24_O_2_S [M+Na]^+^ : 303.1389, found: 303.1389.

The catalytic activity was evaluated for the dual photoredox model reaction of C-N coupling between pyrrolidine and methyl bromobenzoate halides (Figure S8). The mechanism proceeds by two interconnected catalytic cycles whereby visible light (456 nm) irradiation of the CN_x_ generates photoexcited electrons which can be transferred to the coordinated M^II^ species (M = Cu or Ni). This single electron transfer (SET) process reduces the metal to a lower oxidation state, able to sustain the first oxidative addition step by the aryl halide and begin the redox catalytic cycle. This second cycle presumably occurs in solution and requires no light, although a fully heterogeneous mechanism (with the metal always bound to the CN_x_ in form of single atoms) is also possible. Any occurrence of the M^II^ resting state during the catalytic cycle is re-activated by the photoinduced SET. Excessive reduction of the metal, however, may lead to formation of metallic M^0^ eventually causing aggregation into nanoparticles. For this reason, the binding state between CN_x_ and M must be opportunely adjusted, so that the metal species can de-coordinate immediately after the SET, carrying the catalytic reaction in solution phase.

S3. DFT calculation methods.

The spin Hamiltonian parameters, g and A tensors, were predicted based on the equilibrium geometry obtained using the PBE^[18]^ XC functional with 40% Hartree-Fock exchange, augmented by the D3(BJ)^[19]^ dispersion correction. The def2-TZVP^[20]^ basis set was used for light elements (H, C, and N), while the CP(PPP)^[21]^ basis set was employed for Ni and Cu. All the calculations have been performed with the ORCA 6.0.1^[22]^ quantum chemistry software package.

Default SCF criteria have been adopted for geometry optimizations, while tighter SCF convergence (TightSCF) and larger quadrature grid (DefGrid3) have been employed to evaluate the EPR parameters.

Initially, we considered the model proposed by Allasia and coworkers,^[23]^ which features a metal site interacting with two heptazine molecules. However, this simplified model does not account for coordinating nitrogen atoms with different hybridizations. To overcome this limitation, and gain better insight into the possible structure, we explored metal-ligand configurations involving four heptazine molecules and nitrogen atoms spanning different hybridizations.

S4. Figures S1-S30.


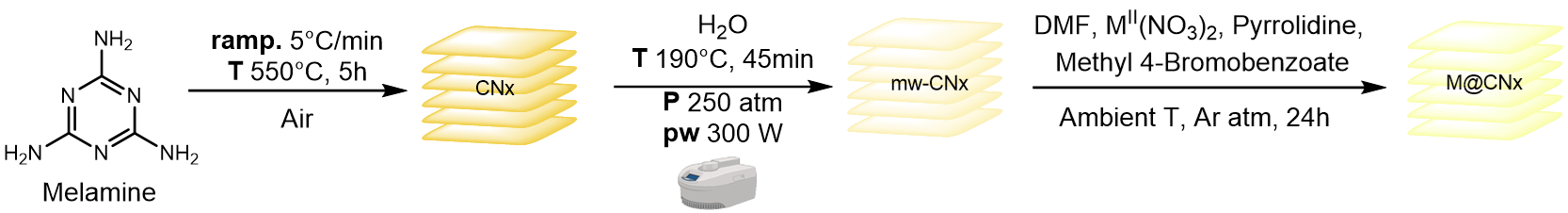


**Figure S1.** schematic representation of the three step synthetic procedure for the metal functionalized materials. M: Ni or Cu.


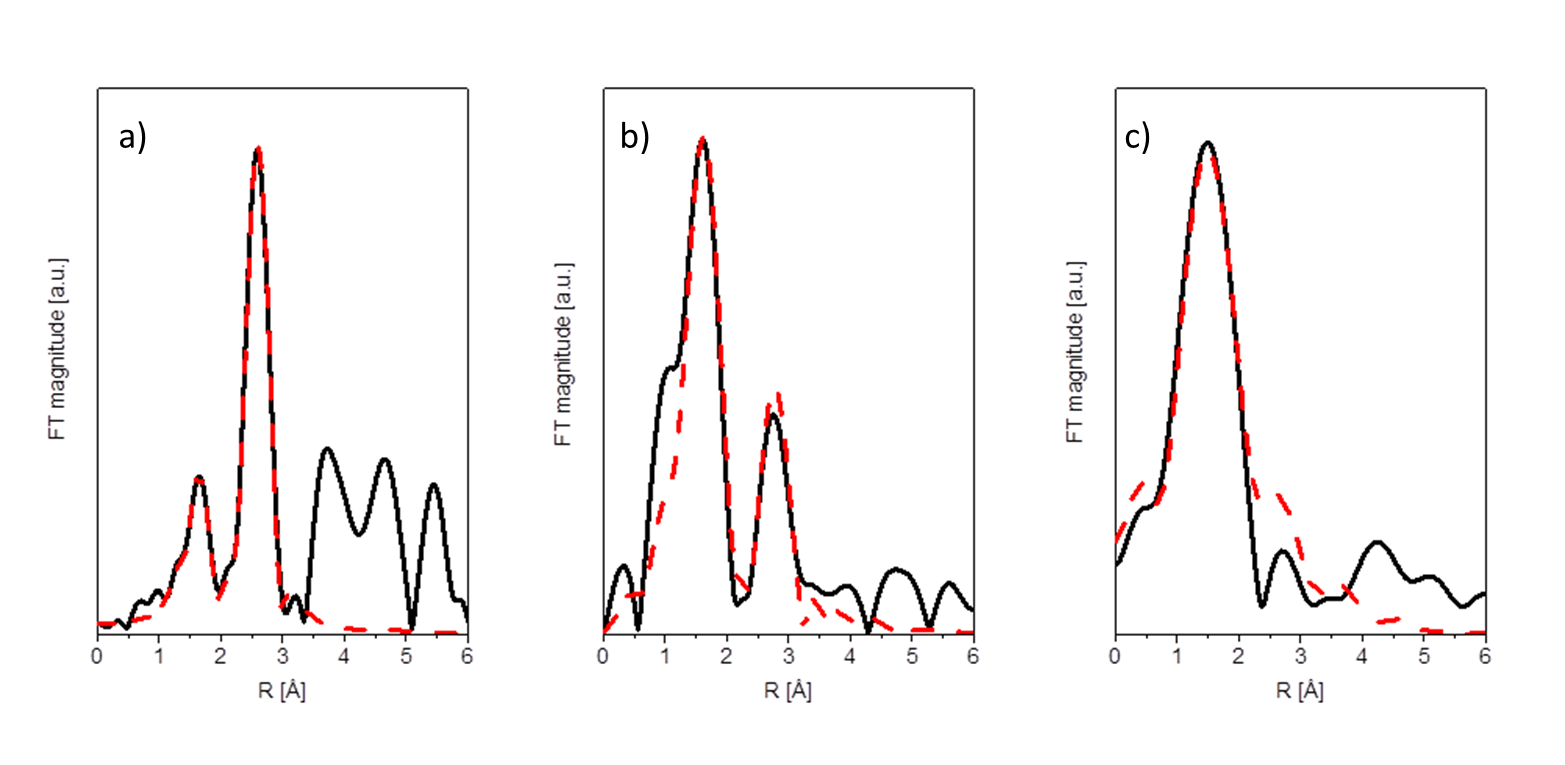


**Figure S2.** Fourier transform magnitude of the Ni K-edge EXAFS spectra of the a) *k^3^*-weighted reference NiO b) *k^2^*-weighted as-prepared catalyst and c) *k*-weighted H_2_-reduced catalyst, a) calculated in the *k*-range of 3−13 Å^−1^, b) in the range of 3−9 Å^−^1, c) in range of 3−8 Å^−^1. Experiment (black line); best-fit EXAFS model (dashed red line) calculated in the *R*-range from 1.2 to 3.2 Å in the case of the NiO and as-prepared catalyst and in the *R*-range from 1.2 to 2.2 Å in the case of the reduced sample. The low-*R* contributions (below 1 Å) in the FT EXAFS in b) and c) are due to the background.


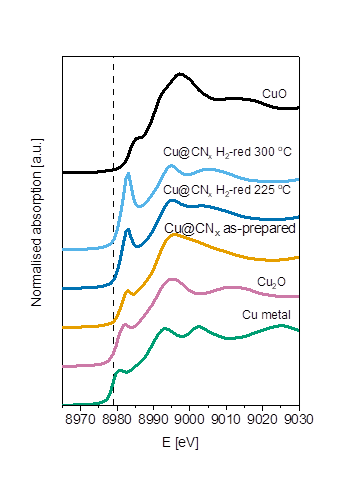
**Figure S3.** Normalized Cu K-edge XANES spectra of the as-prepared and H_2_-reduced Cu@CN_x_ at 225°C and 300°C. Cu reference compounds: Cu metal foil (reference for Cu^0^), crystalline Cu_2_O (reference for Cu^I^) and crystalline CuO (reference for Cu^II^) are shown for comparison. Vertical line is placed at the K-edge of Cu metal foil (8979 eV) to facilitate the comparison of the Cu K-edge edge shifts.

**
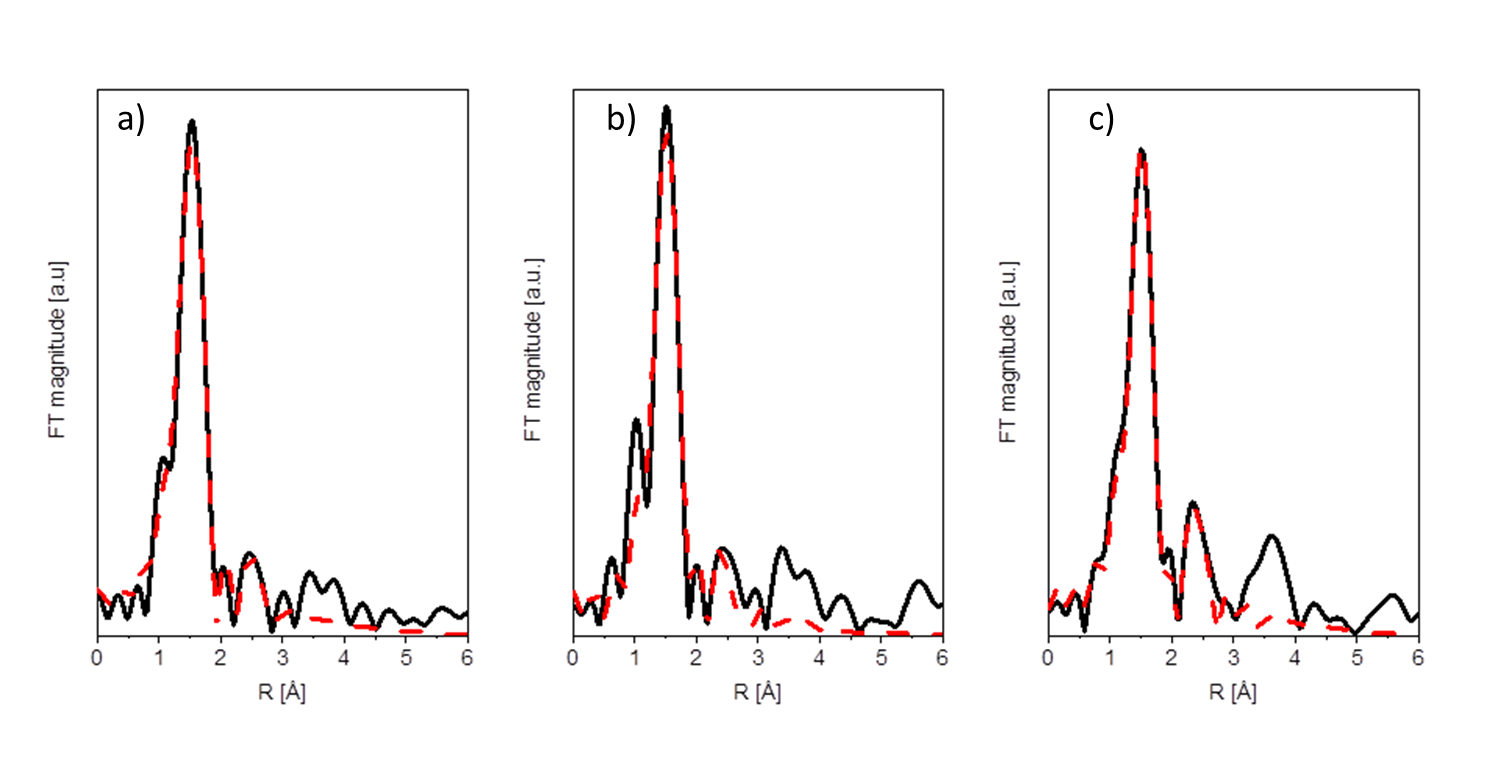
Figure S4.** Fourier transform magnitude of k^3^-weighted Cu EXAFS spectra of the as-prepared (a) and reduced Cu@CN_x_ catalyst in H_2_ atmosphere at 225 °C (b) and 300 °C (c), calculated in the k-range of 3–13 Å^-1^ and R-range of 1.2–2.8 Å. Experiment (black solid line), best-fit EXAFS model (dashed red line).


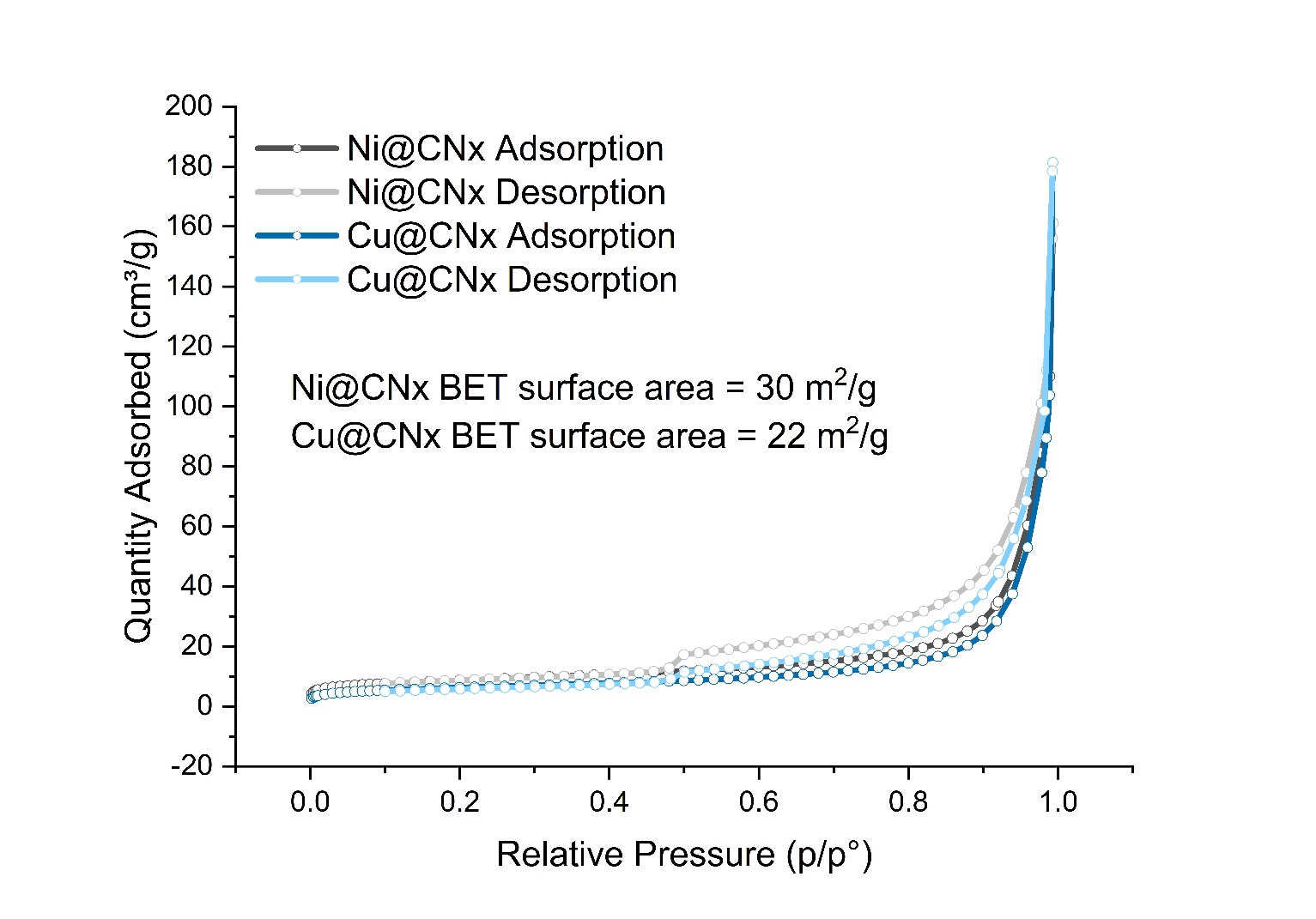


**Figure S5.** N_2_ adsorption/desorption isotherms of Ni@CN_x_ (grey) and Cu@CN_x_ (blue).


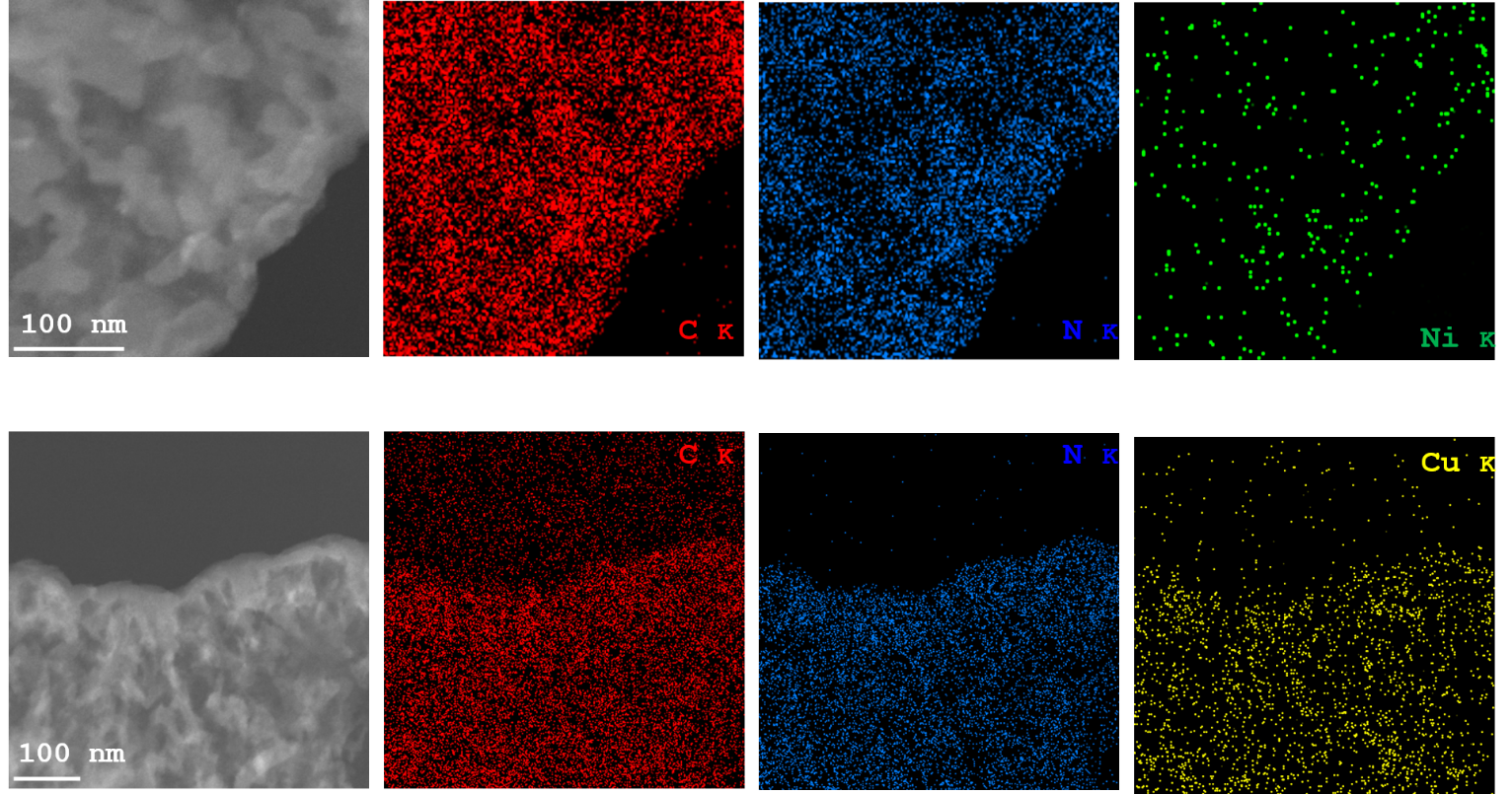


**Figure S6.** STEM-HAADF images with the corresponding EDS maps Ni@CN_x_ (top) and Cu@CN_x_ (bottom).


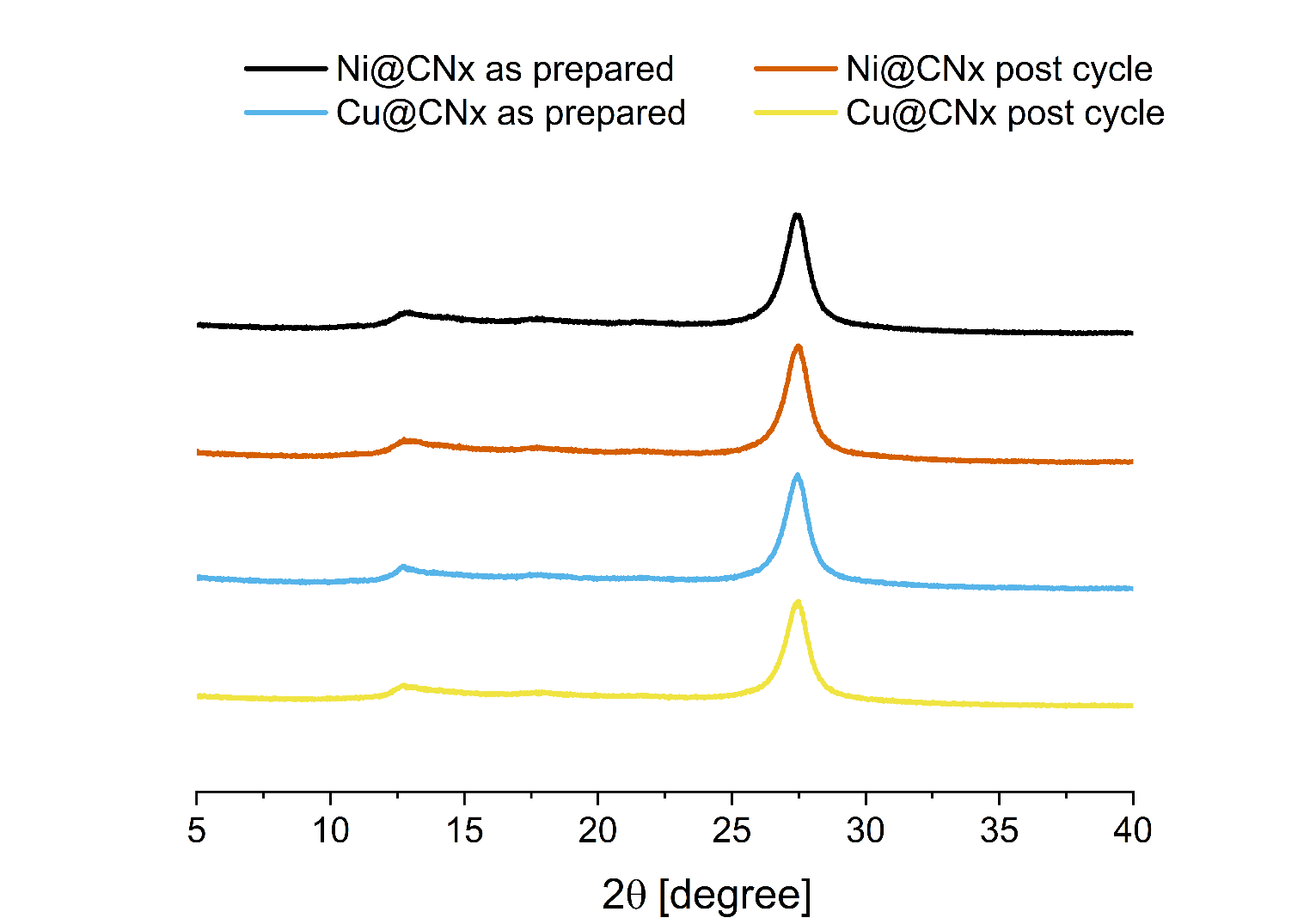


**Figure S7.** Comparison between the XRD diffraction patterns of Ni@CN_x_ as prepared (black), Cu@CN_x_ as prepared (blue) and the same powders after reduction in H_2_ followed by reoxidation in O_2_ (respectively orange and yellow)

**Figure S8.** Schematic representation of the photoredox reaction and reaction set-up.


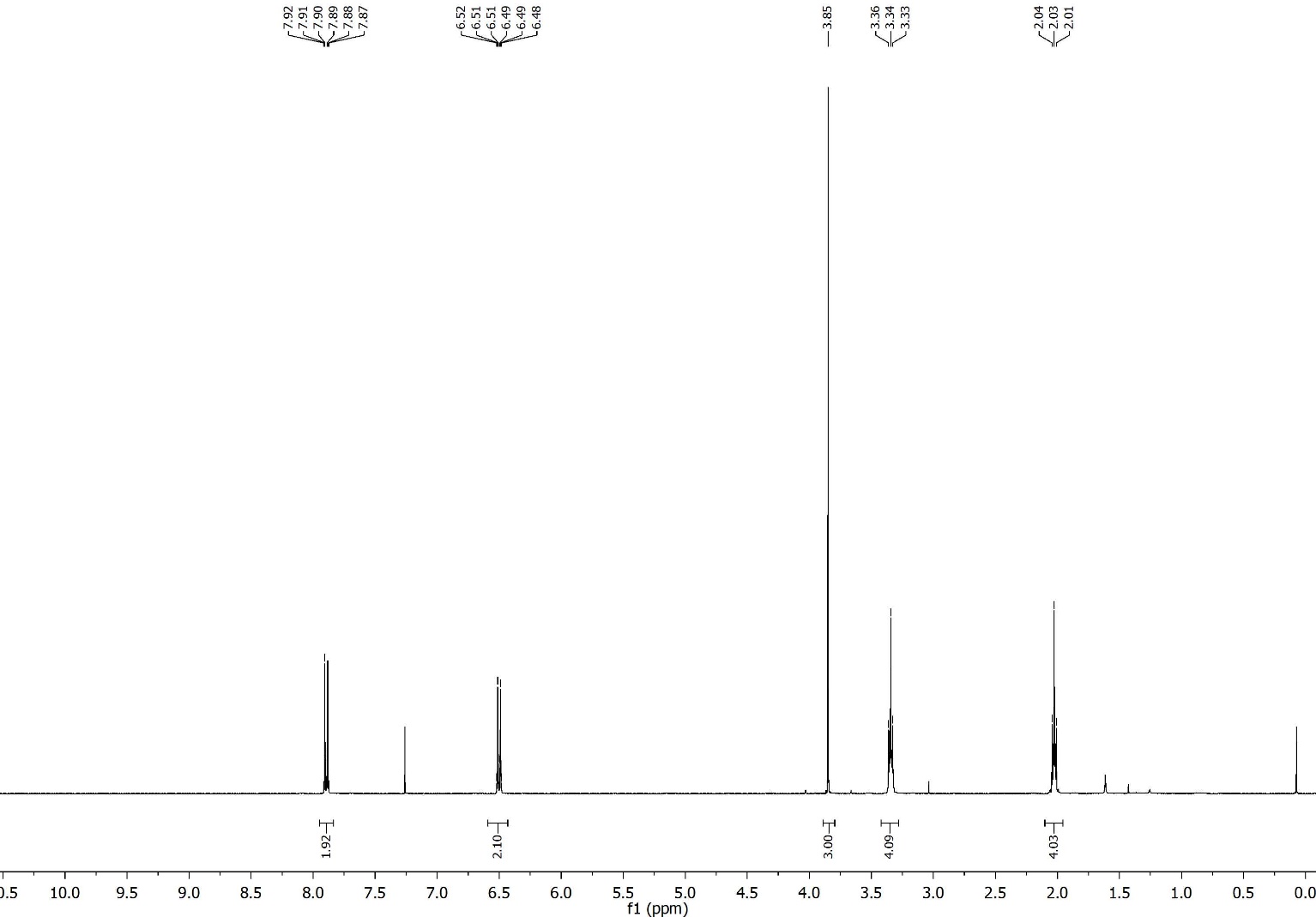

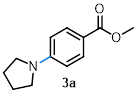


**Figure S9.** ^1^H NMR Methyl 4-(pyrrolidin-1-yl)benzoate **3a** referenced at 7.26 ppm with CDCl_3_ peak.


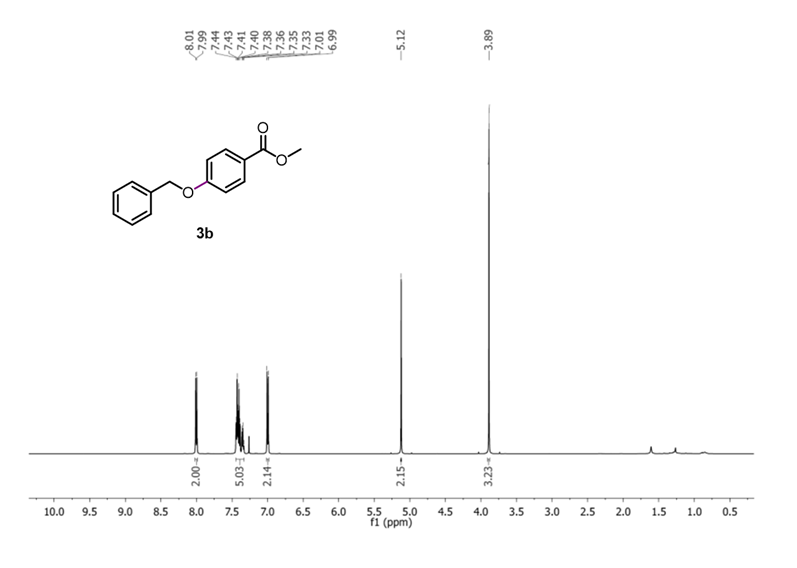


**Figure S10.** ^1^H NMR 4-Benzyloxy-benzoic acid methyl ester **3b** referenced at 7.26 ppm with CDCl_3_ peak.


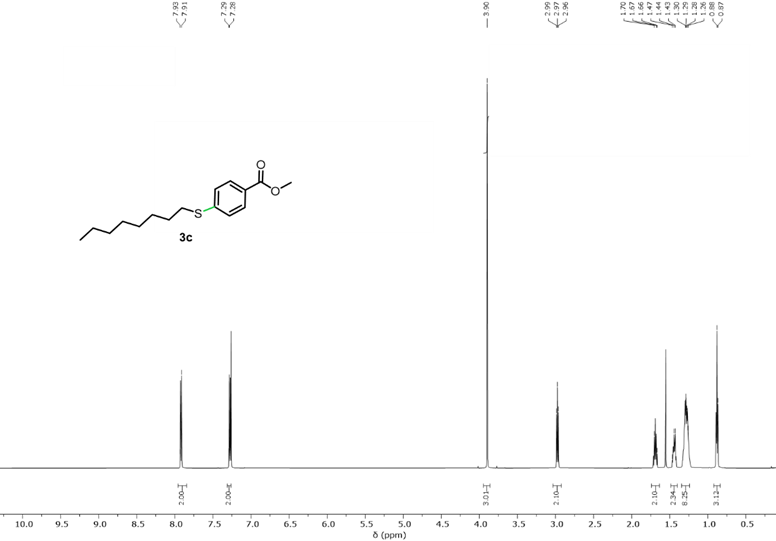


**Figure S11.** ^1^H NMR Methyl 4-(octylthio)benzoate **3c** referenced at 7.26 ppm with CDCl_3_ peak.


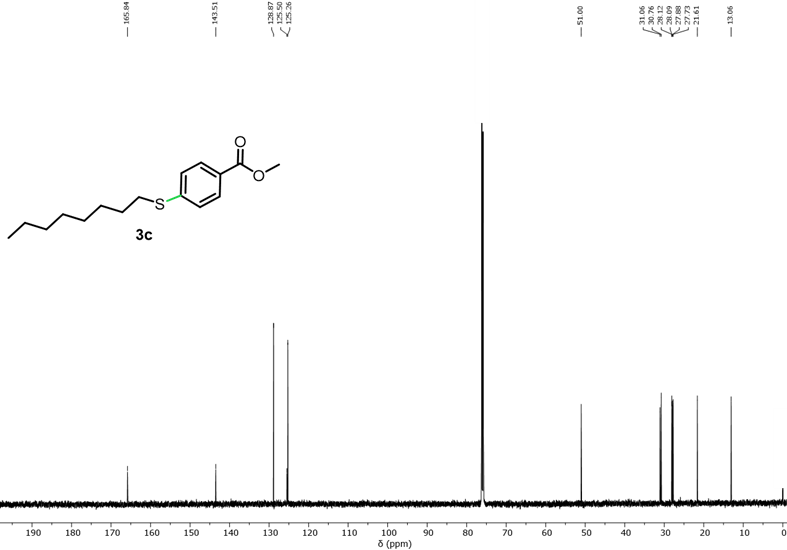


**Figure S12.** ^13^C NMR Methyl 4-(octylthio)benzoate **3c**.

**Figure S13.** Experimental (black) and simulated (yellow) X-band CW EPR spectrum of Ni^I^@CN_x_. The experimental spectrum was recorded at 77 K.

**Figure S14.** Experimental (black) and simulated (yellow) Q-band CW EPR spectrum of of Ni^I^@CN_x_. The deconvolution of the simulated spectrum in the two species (Table 1 main text) is shown. The experimental spectrum was recorded at 10 K.

**Figure S15.** Experimental (black) and simulated (yellow) X-band CW EPR spectrum of Cu^II^@CN_x_. The experimental spectrum was recorded at 77 K.

**Figure S16.** CW EPR normalized spectra recorded at RT of Cu@CN_x_ with different Cu loading. The bar diagram shows the spectral integrated intensity corresponding to the number of Cu^II^ EPR active ions (purple) compared to the Cu loading as determined by ICP-OES (red).


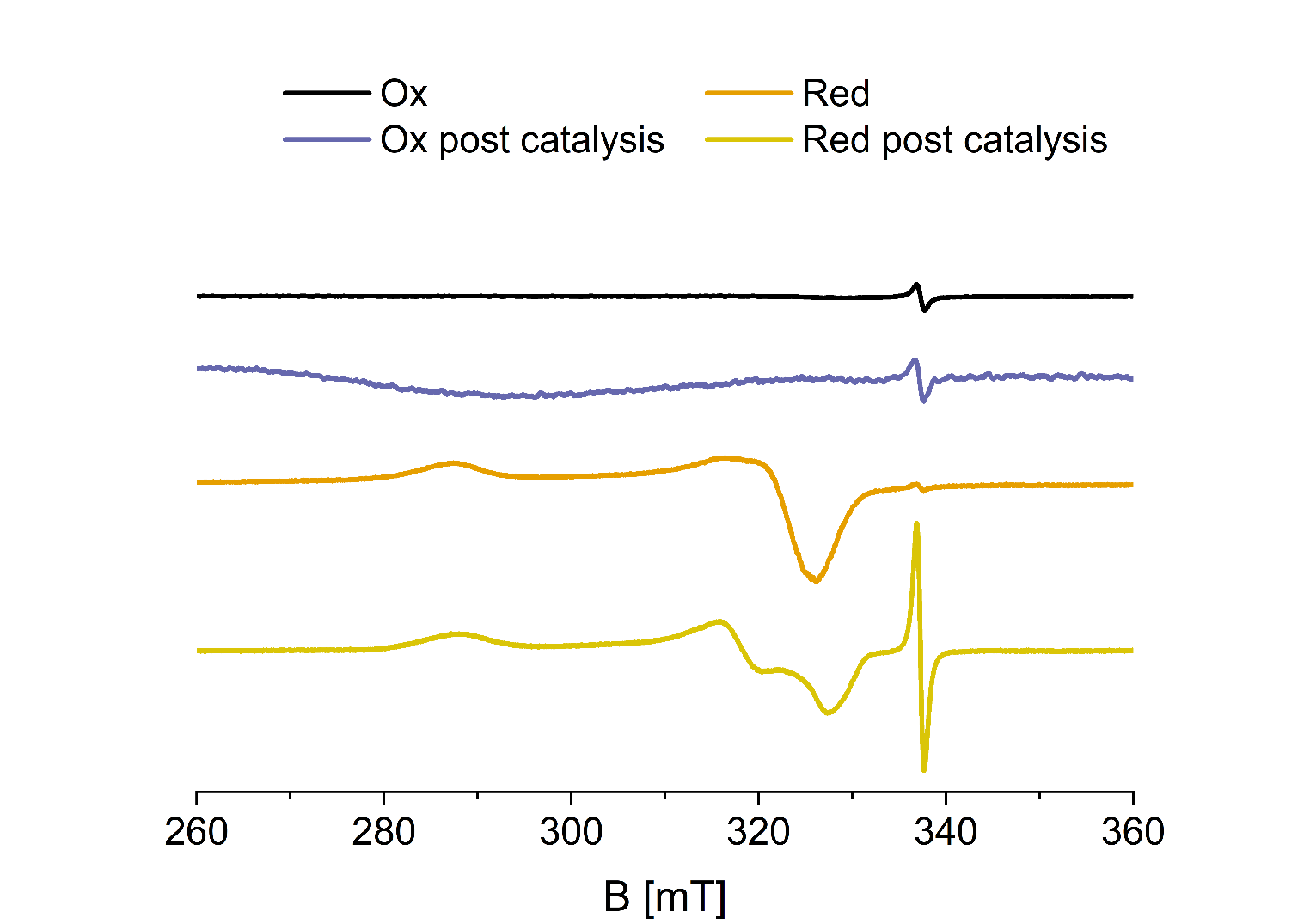


**Figure S17.** Comparison of X-band CW EPR spectrum of Ni@CN_x_ recorded as prepared (black), after reduction in H_2_ (orange), after utilization as catalyst in the reactions described in section S2 (purple), after rereduction in H_2_ (yellow). All spectra were recorded at 77 K.


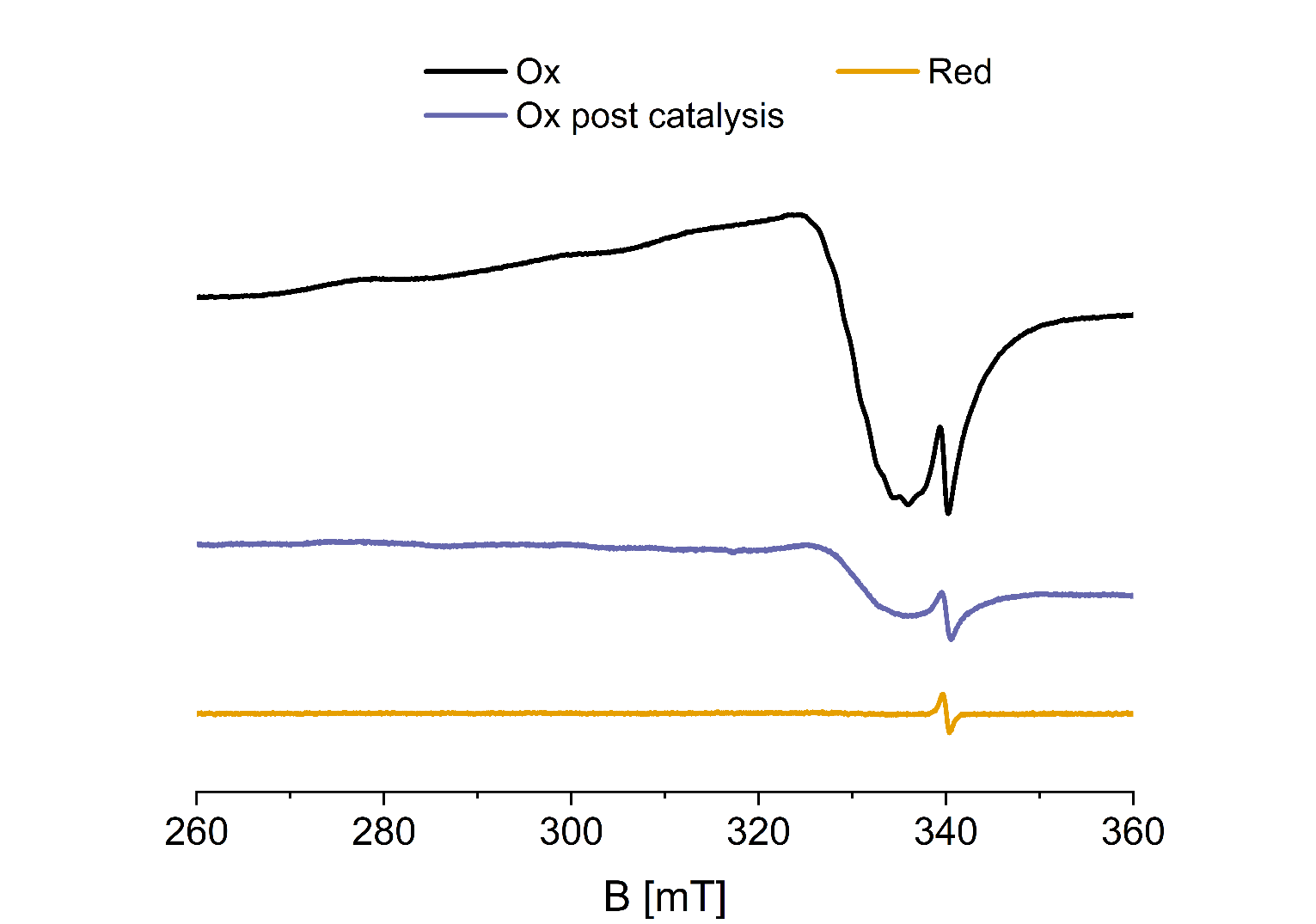


**Figure S18.** Comparison of X-band CW EPR spectrum of Cu@CN_x_ recorded as prepared (black), after reduction in H_2_ (orange), after utilization as catalyst in the reactions described in section S2 (purple). All spectra were recorded at room temperature.


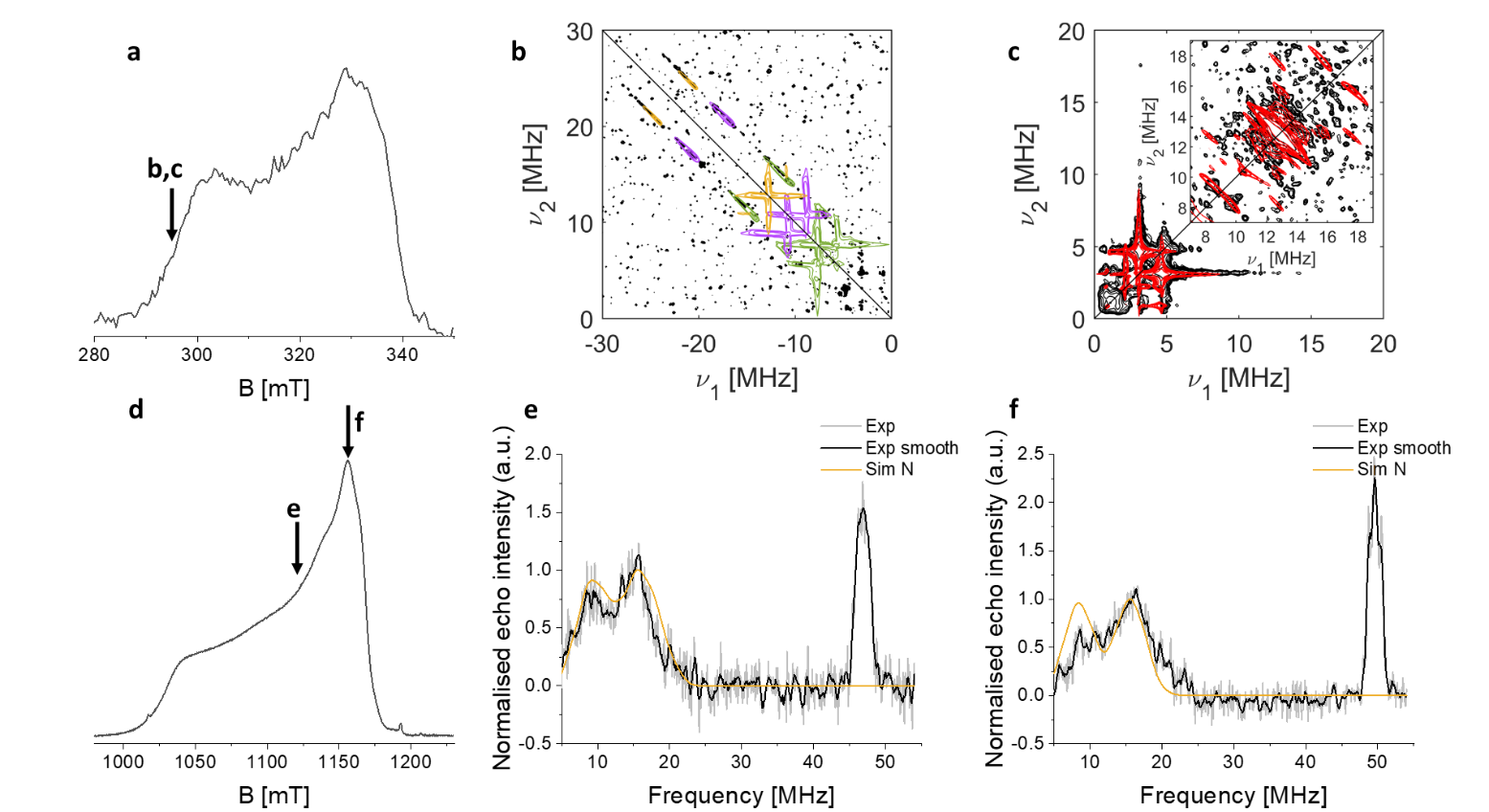


**Figure S19.** a) X-band ESE detected EPR spectrum of Ni^I^@CN_x_. The arrow indicates the magnetic field position at which the HYSCORE experiments were performed; b) Experimental (black) and simulated (colors) X-band Matched HYSCORE spectrum. The colors correspond to the simulation of the different coordinating ^14^N atoms (Table 1 main text); c) X-band standard HYSCORE spectrum black (experiment) red (simulation). Due to the different signal intensities, the proton signal in the inset, is plotted at a different contour level; d) Q-band ESE detected EPR spectrum of Ni^I^@CN_x_. The arrows indicate the magnetic field positions at which the ENDOR experiments were performed; e,f) Q-band Davies ENDOR spectra (black experiment, yellow simulation).

~~
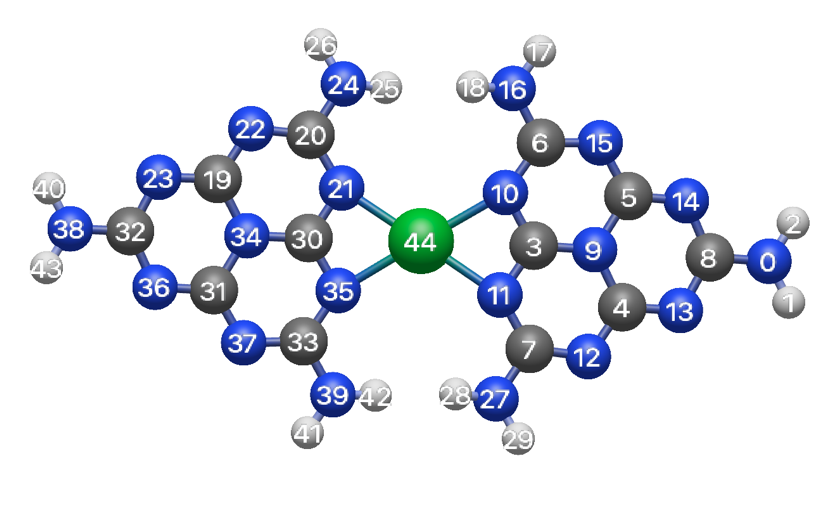
~~

|  | *α* | *β* | *γ* | *A*_x_ | *A*_y_ | *A*_z_ | *a*_iso_ | *e^2^Qq/h* |
| --- | --- | --- | --- | --- | --- | --- | --- | --- |
|  | [degrees] | | | [MHz] | | | | |
| 10N | -76.6 | 39.0 | 94.8 | 19.85 | 16.78 | 16.88 | 17.83 | 2.63 |
| 11N | 76.7 | 40.6 | -103.0 | 19.28 | 16.23 | 16.34 | 17.28 | 2.63 |
| 21N | 79.3 | 38.7 | -103.9 | 19.17 | 16.14 | 16.24 | 17.18 | 2.62 |
| 35N | -79.9 | 34.9 | 97.8 | 20.33 | 17.23 | 17.31 | 18.29 | 2.63 |
| 44Ni | -169.7 | 0.3 | -176.4 | 42.30 | 40.42 | 234.25 | 105.66 | -69.21 |

**Figure S20.** DFT optimized structure of Ni^I^ di-melem complex (4sp^2^) *q*=1, 2*S*+1=2. The calculated EPR parameters are reported in the Table. The Euler angles (*α,β,γ*) define the relative orientation of the **A** tensor with respect to the **g** tensor frame using the “zyz” convention.^[24]^


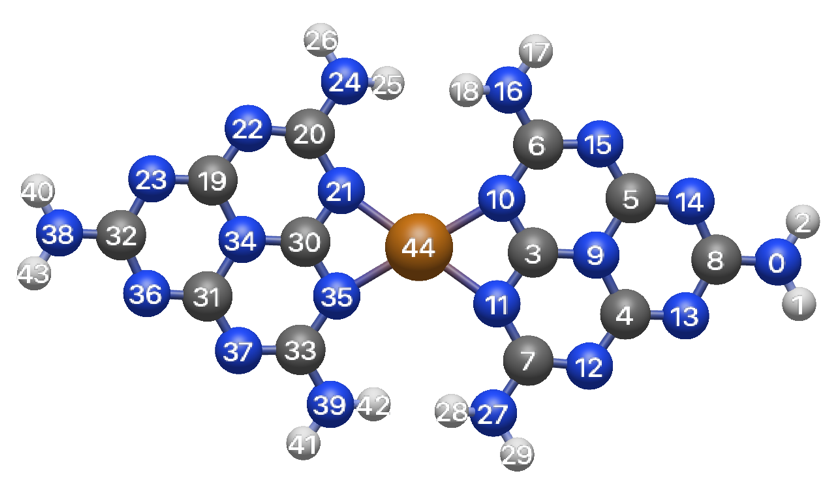


|  | *α* | *β* | *γ* | *A*_x_ | *A*_y_ | *A*_z_ | *a*_iso_ | *e^2^Qq/h* |
| --- | --- | --- | --- | --- | --- | --- | --- | --- |
|  | [degrees] | | | [MHz] | | | | |
| 10N | -8.9 | 2.1 | 19.2 | 36.32 | 44.96 | 36.73 | 39.34 | 2.53 |
| 11N | -170.9 | 1.7 | 150.2 | 36.25 | 44.89 | 36.66 | 39.26 | 2.53 |
| 21N | -179.6 | 0.8 | 158.9 | 36.43 | 45.09 | 36.84 | 39.45 | 2.53 |
| 35N | 8.0 | 2.1 | 2.4 | 36.65 | 45.35 | 37.07 | 39.69 | 2.53 |
| 44Cu | 118.2 | 0.6 | -94.7 | -3.75 | -6.37 | -637.94 | -216.02 | 96.19 |

**Figure S21.** DFT optimized structure of Cu^II^ di-melem complex (4sp^2^) *q*=2 2*S*+1=2. The calculated EPR parameters are reported in the Table. The Euler angles (*α,β,γ*) define the relative orientation of the **A** tensor with respect to the **g** tensor frame using the “zyz” convention.^[24]^


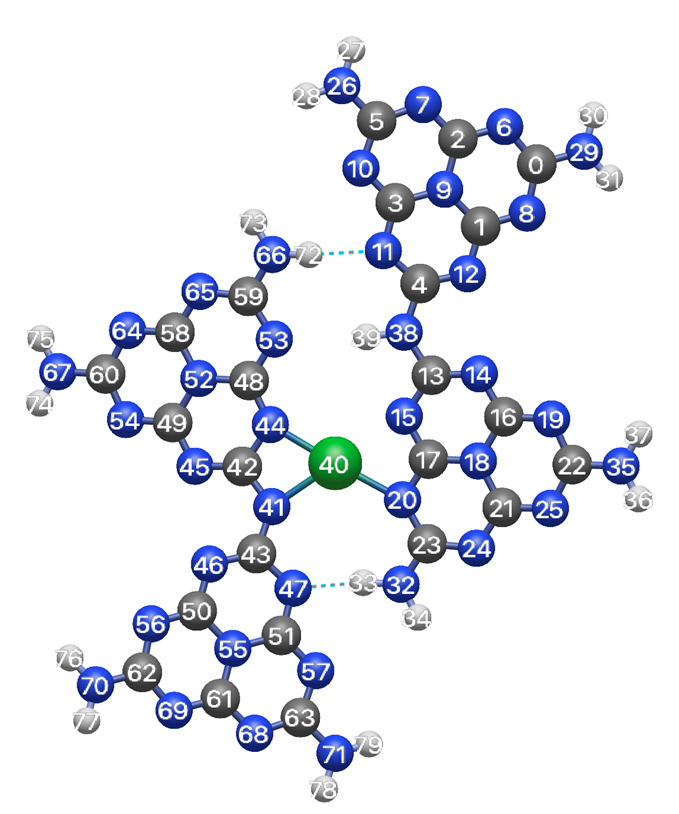


|  | *α* | *β* | *γ* | *A*_x_ | *A*_y_ | *A*_z_ | *a*_iso_ | *e^2^Qq/h* |
| --- | --- | --- | --- | --- | --- | --- | --- | --- |
|  | [degrees] | | | [MHz] | | | | |
| 15N | -76.4 | 7.2 | 81.3 | 22.18 | 19.37 | 19.47 | 20.34 | 2.57 |
| 18N | 82.90 | 6.30 | -58.00 | -0.11 | -0.37 | -0.31 | -0.26 | -2.60 |
| 20N | -67.1 | 9.4 | 110.4 | 16.51 | 13.59 | 13.78 | 14.63 | 2.57 |
| 41N | -115.1 | 15.9 | 116.4 | 24.78 | 20.24 | 20.51 | 21.85 | 2.80 |
| 44N | -15.2 | 5.5 | -29.7 | 17.12 | 20.35 | 17.39 | 18.29 | -2.40 |
| 55N | 174.9 | 3.6 | 145.9 | 0.06 | -0.05 | -0.02 | 0.00 | -2.64 |
| 71N | -168.4 | 17.2 | -167.5 | -0.01 | 0.03 | -0.02 | 0.00 | -4.36 |
| 32N | -7.5 | 6.5 | 6.3 | -0.01 | 0.37 | -0.15 | 0.07 | -2.88 |
| 33H | 177.7 | 1 | -166.1 | -2.15 | 4.81 | -2.61 | 0.01 | 0.20 |
| 39H | 152.9 | 0.9 | -116.7 | -1.89 | 4.03 | -2.27 | -0.04 | 0.24 |
| 40Ni | -143.4 | 0.9 | 131 | 39.04 | 36.33 | 230.45 | 101.94 | -59.23 |

**Figure S22.** DFT optimized structure of Ni^I^ tetra-melem complex (3sp^2^_sp^3^) *q*=0 2*S*+1=2. The calculated EPR parameters are reported in the Table. The Euler angles (*α,β,γ*) define the relative orientation of the **A** tensor with respect to the **g** tensor frame using the “zyz” convention.^[24]^


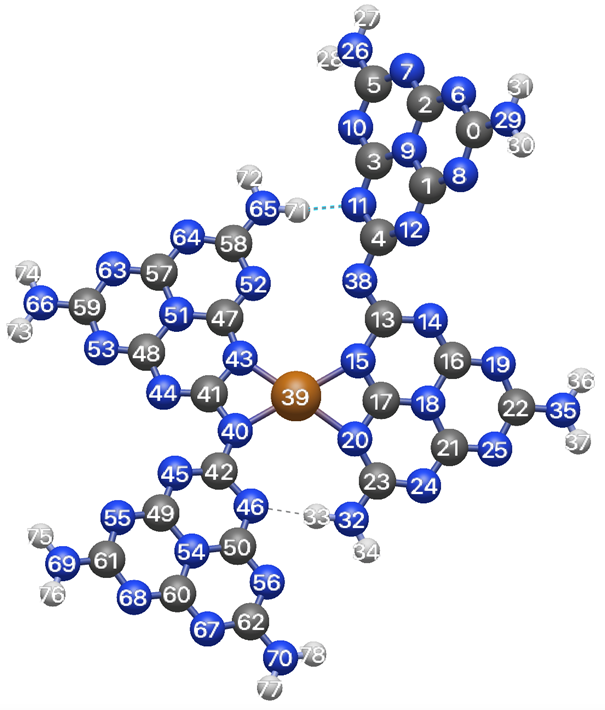


|  | *α* | *β* | *γ* | *A*_x_ | *A*_y_ | *A*_z_ | *a*_iso_ | *e^2^Qq/h* |
| --- | --- | --- | --- | --- | --- | --- | --- | --- |
|  | [degrees] | | | [MHz] | | | | |
| 15N | 88.80 | 17.10 | -53.50 | 41.53 | 33.18 | 33.69 | 36.13 | 2.21 |
| 18N | 107.40 | 8.20 | -147.90 | -0.59 | -0.36 | -0.72 | -0.55 | -2.94 |
| 20N | 50.80 | 11.00 | -73.60 | 35.99 | 43.35 | 36.30 | 38.55 | 2.64 |
| 32N | 128.60 | 12.30 | -100.40 | -0.27 | 0.06 | -0.43 | -0.21 | -3.32 |
| 40N | -112.90 | 17.50 | 144.20 | 48.69 | 37.77 | 38.62 | 41.69 | 2.73 |
| 43N | -149.60 | 12.60 | 129.20 | 43.34 | 53.31 | 44.08 | 46.91 | 2.40 |
| 39Cu | -130.00 | 0.60 | 137.60 | 9.03 | -28.32 | -646.67 | -221.99 | 87.48 |
| 33H | 108.90 | 5.7 | -66.90 | -1.60 | 3.82 | -2.16 | 0.02 | 0.23 |

**Figure S23.** DFT optimized structure of Cu^II^ tetra-melem complex (3sp^2^_sp^3^) *q*=0 2*S*+1=2. The calculated EPR parameters are reported in the Table. The Euler angles (*α,β,γ*) define the relative orientation of the **A** tensor with respect to the **g** tensor frame using the “zyz” convention.^[24]^


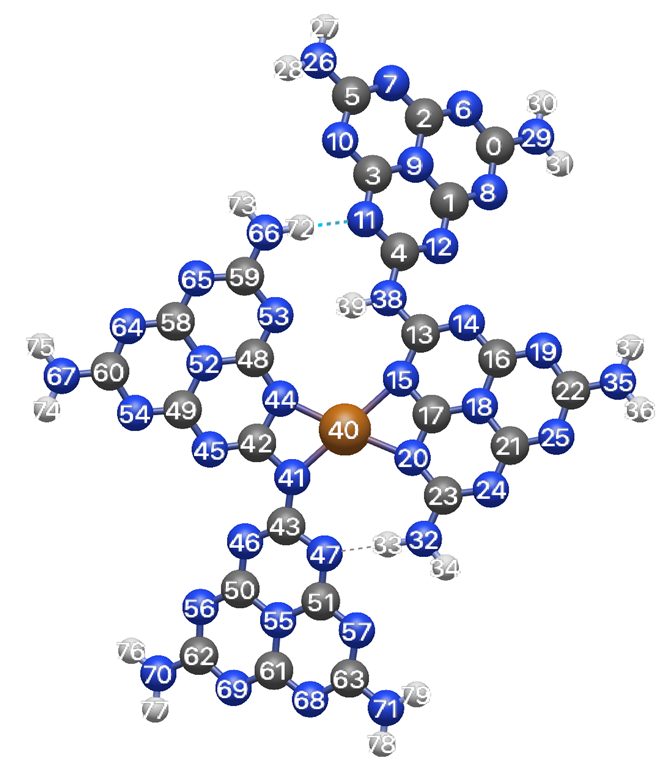


|  | *α* | *β* | *γ* | *A*_x_ | *A*_y_ | *A*_z_ | *a*_iso_ | *e^2^Qq/h* |
| --- | --- | --- | --- | --- | --- | --- | --- | --- |
|  | [degrees] | | | [MHz] | | | | |
| 15N | -87.5 | 9.2 | 131.0 | 38.90 | 32.31 | 32.65 | 34.62 | 2.54 |
| 20N | -152.4 | 14.5 | 137.4 | 32.72 | 39.83 | 33.08 | 35.21 | 2.52 |
| 32N | -91.7 | 7.2 | 128.4 | -0.22 | 0.11 | -0.35 | -0.15 | -2.70 |
| 41N | 72.6 | 17.5 | -32.5 | 54.70 | 41.37 | 42.42 | 46.16 | 2.67 |
| 44N | 45.8 | 5.9 | -57.5 | 45.52 | 55.89 | 46.24 | 49.22 | 2.43 |
| 55N | -85.7 | 43.8 | 79.6 | 0.05 | -0.06 | -0.09 | -0.03 | -2.58 |
| 71N | -77.8 | 9.1 | 47.8 | 0.03 | -0.01 | -0.02 | 0.00 | -4.13 |
| 33H | -162.8 | 5.0 | 122.1 | 3.91 | -1.67 | -2.28 | -0.01 | 0.20 |
| 39H | 176.7 | 2.3 | 163.5 | 3.84 | -1.67 | -2.27 | -0.03 | 0.23 |
| 40Cu | 28.3 | 0.5 | -19.7 | -10.99 | -35.09 | -664.15 | -236.74 | 86.50 |

**Figure S24.** DFT optimized structure of Cu^II^ tetra-melem complex (3sp^2^_sp^3^) *q*=+1 2*S*+1=2. The calculated EPR parameters are reported in the Table. The Euler angles (*α,β,γ*) define the relative orientation of the **A** tensor with respect to the **g** tensor frame using the “zyz” convention.^[24]^


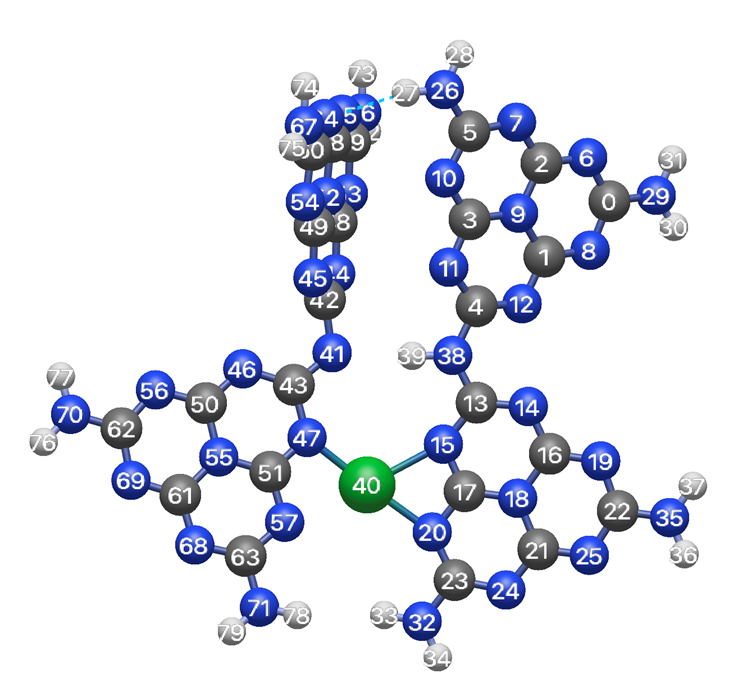


|  | *α* | *β* | *γ* | *A*_x_ | *A*_y_ | *A*_z_ | *a*_iso_ | *e^2^Qq/h* |
| --- | --- | --- | --- | --- | --- | --- | --- | --- |
|  | [degrees] | | | [MHz] | | | | |
| 15N | 42.30 | 15.50 | -28.00 | 25.46 | 21.86 | 22.04 | 23.12 | -2.80 |
| 18N | 57.90 | 9.90 | -28.80 | -0.14 | -0.42 | -0.37 | -0.31 | -2.59 |
| 20N | -50.70 | 9.10 | 17.00 | 8.28 | 10.47 | 8.37 | 9.04 | 2.63 |
| 32N | 6.20 | 19.40 | -2.90 | 0.03 | 0.43 | -0.06 | 0.13 | -4.19 |
| 41N | -12.6 | 13.9 | 24.9 | -0.02 | 0.44 | -0.08 | 0.12 | 2.62 |
| 47N | -169.90 | 24.0 | 138.30 | 8.35 | 11.24 | 8.56 | 9.38 | 2.33 |
| 57N | -98.20 | 32.10 | 116.10 | 21.94 | 19.09 | 19.13 | 20.05 | 2.72 |
| 33H | 100.20 | 8.80 | -84.40 | -2.10 | 4.60 | -2.46 | 0.01 | 0.29 |
| 39H | 72.40 | 4.40 | -31.00 | -1.98 | 4.18 | -2.39 | -0.06 | 0.21 |
| 78H | 177.30 | 13.3 | 145.90 | 2.94 | -1.35 | -1.56 | 0.01 | 0.29 |
| 40Ni | 79.9 | 0.3 | -93 | -19.53 | -37.93 | 157.21 | 33.25 | -68.66 |

**Figure S25.** DFT optimized structure of Ni^I^ tetra-melem complex (4sp^2^) q=0 2S+1=2. The calculated EPR parameters are reported in the Table. The Euler angles (*α,β,γ*) define the relative orientation of the **A** tensor with respect to the **g** tensor frame using the “zyz” convention.^[24]^


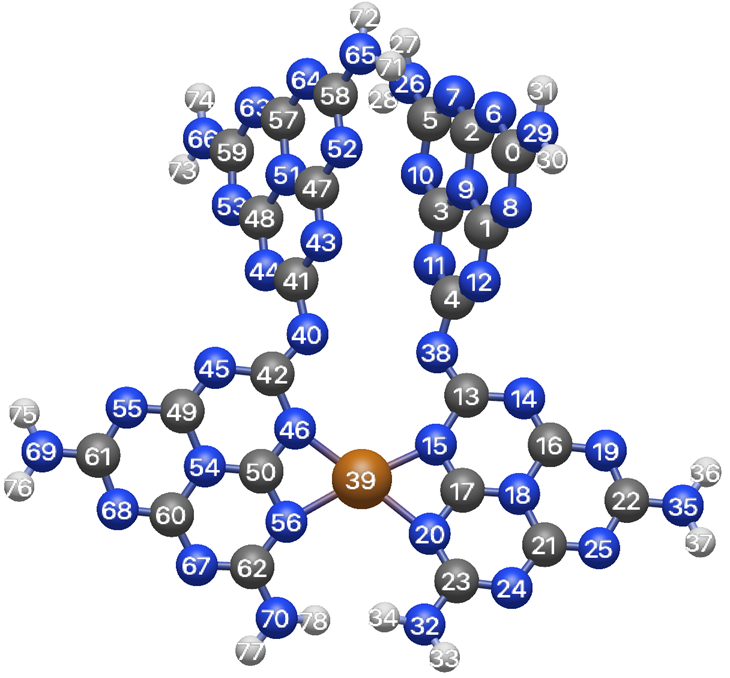


|  | *α* | *β* | *γ* | *A*_x_ | *A*_y_ | *A*_z_ | *a*_iso_ | *e^2^Qq/h* |
| --- | --- | --- | --- | --- | --- | --- | --- | --- |
|  | [degrees] | | | [MHz] | | | | |
| 15N | 118.2 | 15.9 | -158.3 | 44.37 | 33.83 | 34.55 | 37.59 | 1.97 |
| 18N | 60.6 | 8.6 | -22.2 | -0.51 | -0.22 | -0.69 | -0.47 | -2.93 |
| 20N | 159 | 6 | -142.8 | 36.72 | 43.72 | 36.99 | 39.14 | 2.67 |
| 32N | 130.7 | 14.6 | -163.3 | -0.36 | -0.04 | -0.48 | -0.29 | -4.27 |
| 46N | -35.6 | 14.6 | 54.5 | 35.34 | 45.96 | 36.1 | 39.13 | 1.98 |
| 56N | -82.4 | 11.6 | 45.3 | 42.54 | 35.71 | 35.97 | 38.07 | 2.67 |
| 34H | 174.9 | 15.5 | -131.3 | 3.47 | -1.55 | -1.99 | -0.02 | 0.29 |
| 78H | 6.5 | 16.4 | 17.4 | 3.54 | -1.59 | -2.04 | -0.03 | 0.29 |
| 39Cu | -68.6 | 0.1 | 51.2 | 8.58 | -14.85 | -628.75 | -211.67 | 93.68 |

**Figure S26.** DFT optimized structure of Cu^II^ tetra-melem complex (4sp^2^) q=0 2S+1=2. The calculated EPR parameters are reported in the Table. The Euler angles (*α,β,γ*) define the relative orientation of the **A** tensor with respect to the **g** tensor frame using the “zyz” convention.^[24]^


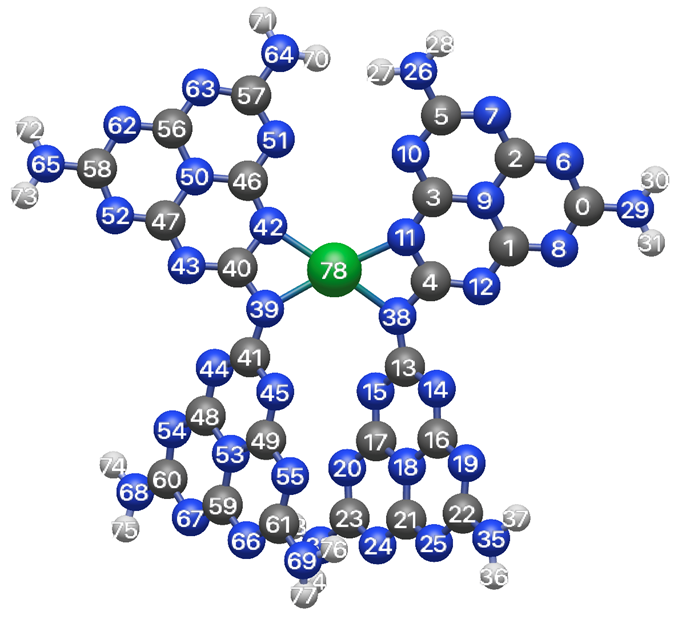


|  | *α* | *β* | *γ* | *A*_x_ | *A*_y_ | *A*_z_ | *a*_iso_ | *e^2^Qq/h* |
| --- | --- | --- | --- | --- | --- | --- | --- | --- |
|  | [degrees] | | | [MHz] | | | | |
| 11N | -69 | 18.8 | 89.6 | 19.64 | 16.61 | 16.89 | 17.71 | -2.69 |
| 38N | 69.9 | 29.1 | -100.6 | 15.35 | 12.78 | 12.87 | 13.67 | 2.70 |
| 39N | -72.3 | 27.6 | 103 | 15.7 | 13.1 | 13.19 | 13.99 | 2.70 |
| 42N | 77.8 | 20.6 | -98.2 | 19.29 | 16.29 | 16.55 | 17.38 | -2.67 |
| 50N | 116.1 | 23 | -130.2 | 1.13 | 0.83 | 0.68 | 0.88 | -2.77 |
| 64N | 106.3 | 10.2 | -88.5 | -0.03 | 0.05 | -0.06 | -0.02 | -4.35 |
| 78Ni | 30.1 | 0.8 | 1.7 | 30.81 | 29.14 | 217.27 | 92.41 | -65.21 |

**Figure S27.** DFT optimized structure of Ni^I^ tetra-melem complex (2sp^2^_2sp^3^) *q*=-1 2*S*+1=2. The calculated EPR parameters are reported in the Table. The Euler angles (*α,β,γ*) define the relative orientation of the **A** tensor with respect to the **g** tensor frame using the “zyz” convention.^[24]^


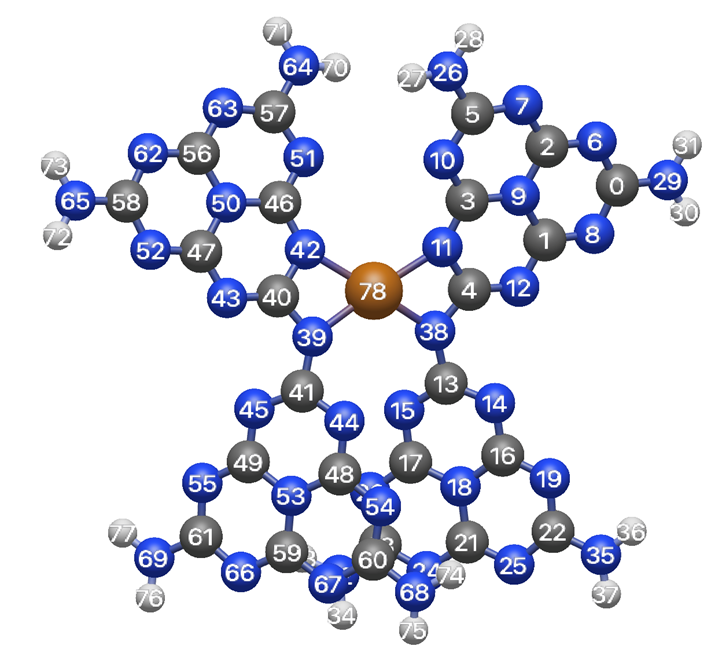


|  | *α* | *β* | *γ* | *A*_x_ | *A*_y_ | *A*_z_ | *a*_iso_ | *e^2^Qq/h* |
| --- | --- | --- | --- | --- | --- | --- | --- | --- |
|  | [degrees] | | | [MHz] | | | | |
| 11N | -155.6 | 19.4 | -166.5 | 37.21 | 44.67 | 37.74 | 39.87 | 2.39 |
| 38N | 136.8 | 9.5 | -138.0 | 34.90 | 45.59 | 35.69 | 38.73 | 2.64 |
| 39N | 37.6 | 9.1 | 3.4 | 34.34 | 45.1 | 35.12 | 38.19 | 2.64 |
| 42N | -28.5 | 19.8 | 30.2 | 37.90 | 45.43 | 38.44 | 40.59 | 2.39 |
| 50N | 5.0 | 14.0 | 1.8 | 1.1 | 1.7 | 0.9 | 1.24 | -2.84 |
| 64N | 17.2 | 11.8 | 16.8 | 0.1 | 0.0 | -0.1 | -0.01 | -4.16 |
| 78Cu | -152.3 | 0.3 | 110.4 | -0.7 | -13.96 | -643.39 | -219.35 | 90.06 |

**Figure S28.** DFT optimized structure of Cu^II^ tetra-melem complex (2sp^2^_2sp^3^) *q*=0 2*S*+1=2. The calculated EPR parameters are reported in the Table. The Euler angles (*α,β,γ*) define the relative orientation of the **A** tensor with respect to the **g** tensor frame using the “zyz” convention.^[24]^

**Figure S29.** Normalized Ni K-edge XANES spectra a) and Fourier transform magnitude of the k^2^-weighted Ni K-edge EXAFS spectra, calculated in the k range of 3−11 Å^−1^ b) for the used Ni@CN_x_ catalyst and relevant standards.


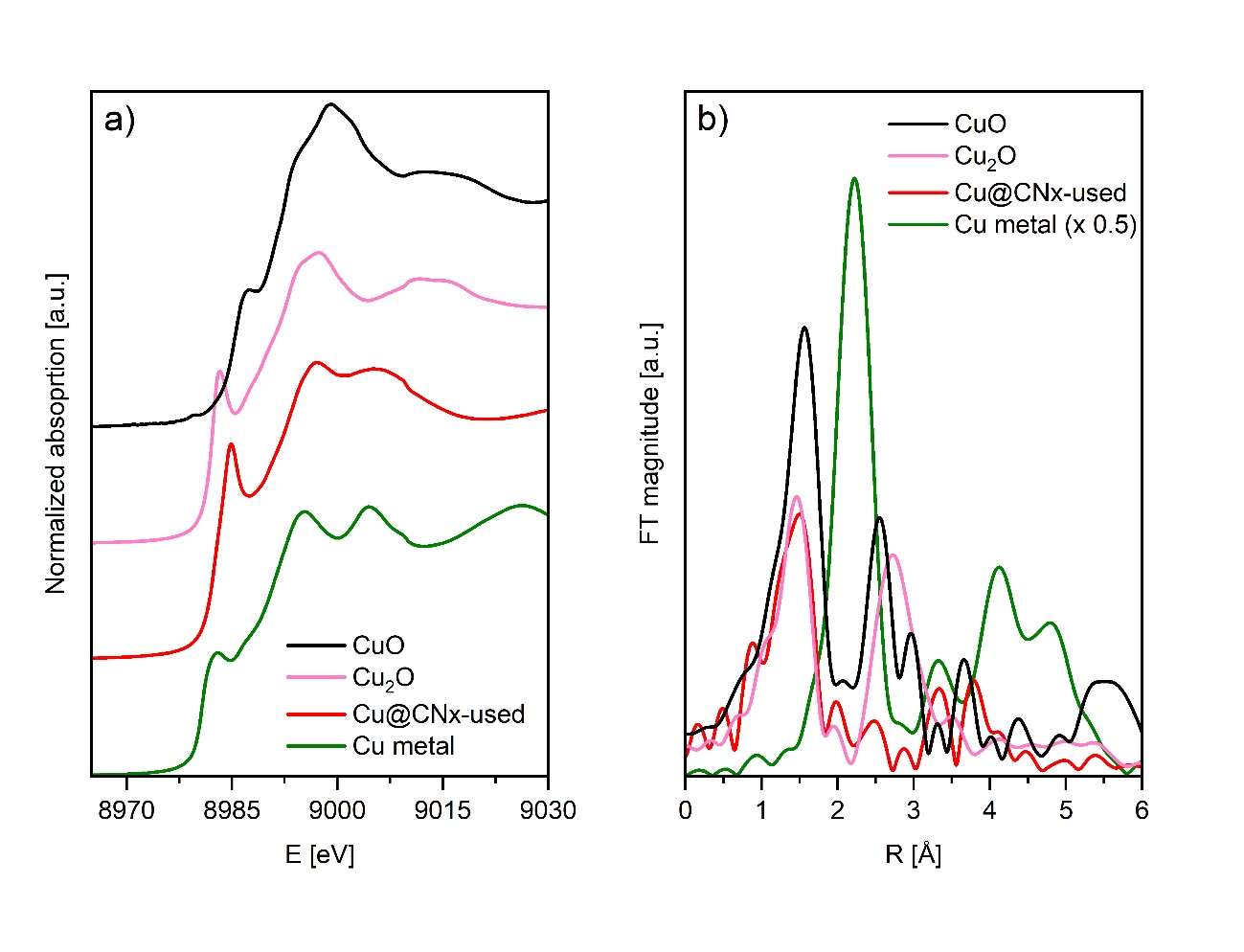


**Figure S30.** Normalized Cu K-edge XANES spectra a) and Fourier transform magnitude of the k^2^-weighted Cu K-edge EXAFS spectra, calculated in the k range of 3−11 Å^−1^ b) for the used Cu@CN_x_ catalyst and relevant standards.


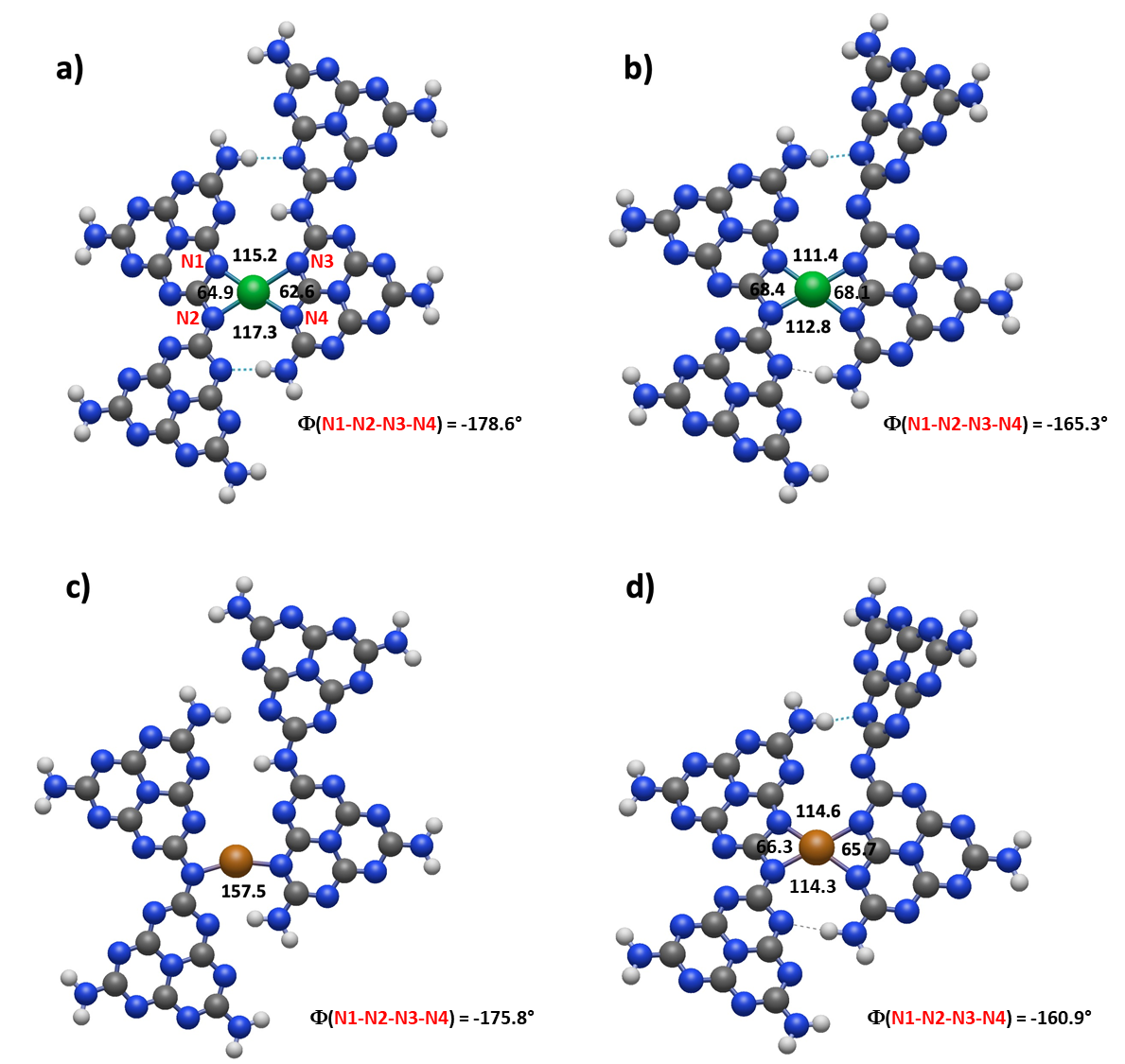


**Figure S31.** DFT optimized angles and dihedral (Φ) angles for the for the proposed 3sp^2^_sp^3^ tetramelem models.

# S5. Tables S1-S5.

| Entry | Metal | Substrate | Time (h) | Yield^a^ (%) | Conversion (%) |
| --- | --- | --- | --- | --- | --- |
| 1 | Ni^II^ | X=Br | 24 | 97±5 (87.5% isolated yield) | 100 |
| 2 | Ni^II^ | X=I | 24 | 98.5±0.1 (89.5% isolated yield) | 100 |
| 3 | Ni^II^ | X=Br | 8 | 17±7 | 24 |
| 4 | Ni^II^ | X=I | 8 | 75±8 | 82 |
| 5 | Cu^II^ | X=Br | 24 | 0 | <5 |
| 6 | Cu^II^ | X=I | 24 | 0 | <5 |
| 7 | Cu^II^ | X=Br | 8 | 0 | <5 |
| 8 | Cu^II^ | X=I | 8 | 0 | <5 |
| Reaction mixture: aryl halide (0.1 mmol), pyrrolidine (0.3 mmol), CN_x_ (2.5 mg/mL), metal(II) salts (5% mol) and DMF (2M).  ^a^NMR yields, using 1,3,5-trimethoxybenzene as internal standard. | | | | | |

**Table S1.** Summary table of the C-N coupling reaction outputs with the corresponding conditions.

**Table S2.** Summary table of the C-O and C-S coupling reactions outputs with the corresponding conditions.

| **Entry** | **Metal** | **Substrate** | **Time (h)** | **Yield^a^ (%)** | **Conversion (%)** |
| --- | --- | --- | --- | --- | --- |
| 1 | Ni^II^ | **2b** | 24 | 70±4 | 94±4 |
| 2 | Cu^II^ | **2b** | 24 | 0 | 10 |
| 3 | Ni^II^ | **2c** | 24 | 58±9 | 77±7 |
| 4 | Cu^II^ | **2c** | 24 | 0 | 40 |
| Reaction mixture: aryl iodine (0.1 mmol), substrate (0.4 mmol), CN_x_ (2.5 mg/mL), metal(II) salts (10% mol), ligand (10% mol), diisopropylamine (0.5 mmol) and DMF (2M).  ^a^NMR yields, using 1,3,5-trimethoxybenzene as internal standard. | | | | | |

**Table S3.** Parameters of nearest neighbour coordination shells around Ni in crystalline NiO, as-prepared and H_2_-reduced Ni@CN_x_ catalysts. Average number of neighbouring atoms (*N*), distance (*R*), and Debye-Waller factor (*σ^2^*). The uncertainty of the last digit is given in parentheses. In the case of crystalline NiO, as-prepared and H_2_-reduced catalysts, the best fit is obtained with the amplitude reduction factor *S_0_^2^*=0.73. The *R*-factor (quality of fit parameter) and the shift of the energy origin (*ΔE_o_* in eV*)* are given in the last column.

| **Ni neigh.** | ***N*** | ***R* [Å]** | ***σ^2^* [Å^2^]** | ***R*-factor** |
| --- | --- | --- | --- | --- |
| NiO crystalline | | | | |
| O | 6 | 2.08(1) | 0.0048(6) | 0.0027  *ΔE_o_* = −3.5(5) eV |
| Ni | 12 | 2.95(1) | 0.0054(2) |  |
| S_0_^2^ = 0.73(4) | | | | |
| as-prepared Ni@CN_x_ | | | | |
| N | 4.8(5) | 2.08(2) | 0.006(2) | 0.009  *ΔE_o_* = 2.6(8) eV |
| S_0_^2^ = 0.73 | | | | |
| H_2_-reduced Ni@CN_x_ | | | | |
| N | 3.6(9) | 2.0(1) | 0.015(9) | 0.015  *ΔE_o_* = 6(10) eV |
| S_0_^2^ = 0.73 | | | | |

**Table S4.** Parameters of the nearest coordination shells around Cu as-prepared and H_2_-reduced at 225 °C and 300 °C Cu@CN_x_ catalysts: average number of neighbor atoms (*N*), distance (*R*) and Debye-Waller factor (*σ^2^*). Uncertainty of the last digit is given in parentheses. A best fit of the catalysts is obtained with the amplitude reduction factor *S_0_^2^*= 0.85. The *R*-factor (quality of fit parameter) and the shift of the energy origin (*ΔE_o_* in eV*)* are given in the last column.

| **Cu neigh.** | ***N*** | ***R* [Å]** | **σ^2^ [Å^2^]** | ***R*-factor** |
| --- | --- | --- | --- | --- |
| as-prepared Cu@CN_x_ | | | | |
| N | 4.5(5) | 1.93(5) | 0.007(1) | 0.012  *ΔE_o_* = 6.2 eV  χ^2^=1200  red. χ^2^=860 |
| C | 1.3(9) | 2.9(1) | 0.009(3) |  |
| H_2_ reduced Cu@CN_x_ at 225 °C | | | | |
| N | 2.8(5) | 1.93(5) | 0.004(1) | 0.016  *ΔE_o_* = 4.4 eV  χ^2^=360  red. χ^2^=384 |
| C | 1.6(8) | 2.9(1) | 0.008(1) |  |
| H_2_ reduced Cu@CN_x_ at 300 °C | | | | |
| N | 2.1(4) | 1.95(5) | 0.004(1) | 0.012  *ΔE_o_* = 7.4(2.3) eV  χ^2^=42  red. χ^2^=45 |
| C | 2.4(7) | 2.9(1) | 0.008(1) |  |

**Table S5.** Parameters of the nearest coordination shells around metal in used Ni@CN_x_ and Cu@CN_x_ catalysts: average number of neighbor atoms (*N*), distance (*R*) and Debye-Waller factor (*σ^2^*). Uncertainty of the last digit is given in parentheses. The *R*-factor (quality of fit parameter) and the shift of the energy origin (*ΔE_o_* in eV*)* are given in the last column.

|  | ***N*** | ***R* [Å]** | **σ^2^ [Å^2^]** | ***R*-factor** |
| --- | --- | --- | --- | --- |
| Used Cu@CN_x_ | | | | |
| N | 2.5(5) | 1.91(1) | 0.004(1) | 0.012  *ΔE_o_* = 2.6 eV  χ^2^=1200  red. χ^2^=860 |
| C | 0.9(6) | 2.9(1) | 0.004(1) |  |
| Used Ni@CN_x_ | | | | |
| N | 4.6(5) | 2.07(2) | 0.002(1) | 0.036  *ΔE_o_* = 4.9 eV  χ^2^=360  red. χ^2^=384 |
| C | 3.4(8) | 3.14(4) | 0.002(1) |  |

# S6. References

[1] M. Marchi, E. Raciti, S. M. Gali, F. Piccirilli, H. Vondracek, A. Actis, E. Salvadori, C. Rosso, A. Criado, C. D’Agostino, L. Forster, D. Lee, A. C. Foucher, R. K. Rai, D. Beljonne, E. A. Stach, M. Chiesa, R. Lazzaroni, G. Filippini, M. Prato, M. Melchionna, P. Fornasiero, “Carbon Vacancies Steer the Activity in Dual Ni Carbon Nitride Photocatalysis” *Adv. Sci.* **2023**, *10*, 2303781.

[2] B. Ravel, M. Newville, “ATHENA, ARTEMIS, HEPHAESTUS: data analysis for X-ray absorption spectroscopy using IFEFFIT” *J. Synchrotron Radiat.* **2005**, *12*, 537–541.

[3] J. J. Rehr, R. C. Albers, S. I. Zabinsky, “High-order multiple-scattering calculations of x-ray-absorption fine structure” *Phys. Rev. Lett.* **1992**, *69*, 3397–3400.

[4] T. Kyômen, R. Yamazaki, M. Itoh, “Valence and spin state of Co and Ni ions and their relation to metallicity and ferromagnetism in LaNi0.5Co0.5O3” *Phys. Rev. B* **2003**, *68*, 104416.

[5] J. Rabeah, J. Radnik, V. Briois, D. Maschmeyer, G. Stochniol, S. Peitz, H. Reeker, C. La Fontaine, A. Brückner, “Tracing Active Sites in Supported Ni Catalysts during Butene Oligomerization by Operando Spectroscopy under Pressure” *ACS Catal.* **2016**, *6*, 8224–8228.

[6] G. Vilé, N. Allasia, S. Xu, S. F. Jafri, E. Borfecchia, L. A. Cipriano, G. Terraneo, S. Tosoni, L. Mino, G. D. Liberto, G. Pacchioni, **2024**, Research Square preprint, DOI: 10.21203/rs.3.rs-4812493/v1.

[7] N. Rossetti, A. Ugolotti, C. Cometto, V. Celorrio, G. Dražić, C. D. Valentin, L. Calvillo, “Insights into the active nickel centers embedded in graphitic carbon nitride for the oxygen evolution reaction” *J. Mater. Chem. A* **2024**, *12*, 6652–6662.

[8] F. W. Lytle, R. B. Greegor, A. J. Panson, “Discussion of x-ray-absorption near-edge structure: Application to Cu in the high-Tc superconductors La1.8Sr0.2CuO4 and YBa2Cu3O7” *Phys. Rev. B* **1988**, *37*, 1550–1562.

[9] C. Maurizio, F. d’Acapito, M. Benfatto, S. Mobilio, E. Cattaruzza, F. Gonella, “Local coordination geometry around Cu and Cu ions in silicate glasses: an X-ray absorption near edge structure investigation” *Eur. Phys. J. B - Condens. Matter Complex Syst.* **2000**, *14*, 211–216.

[10] A. Manceau, A. Matynia, “The nature of Cu bonding to natural organic matter” *Geochim. Cosmochim. Acta* **2010**, *74*, 2556–2580.

[11] X. Dai, Y. Han, H. Jiao, F. Shi, J. Rabeah, A. Brückner, “Aerobic Oxidative Synthesis of Formamides from Amines and Bioderived Formyl Surrogates” *Angew. Chem. Int. Ed.* **2024**, *63*, e202402241.

[12] C. Cometto, A. Ugolotti, E. Grazietti, A. Moretto, G. Bottaro, L. Armelao, C. Di Valentin, L. Calvillo, G. Granozzi, “Copper single-atoms embedded in 2D graphitic carbon nitride for the CO2 reduction” *Npj 2D Mater. Appl.* **2021**, *5*, 63.

[13] P. Schosseler, Th. Wacker, A. Schweiger, “Pulsed ELDOR detected NMR” *Chem. Phys. Lett.* **1994**, *224*, 319–324.

[14] N. Cox, A. Nalepa, W. Lubitz, A. Savitsky, “ELDOR-detected NMR: A general and robust method for electron-nuclear hyperfine spectroscopy?” *J. Magn. Reson.* **2017**, *280*, 63–78.

[15] P. Höfer, A. Grupp, H. Nebenführ, M. Mehring, “Hyperfine sublevel correlation (hyscore) spectroscopy: a 2D ESR investigation of the squaric acid radical” *Chem. Phys. Lett.* **1986**, *132*, 279–282.

[16] S. Stoll, A. Schweiger, “EasySpin, a comprehensive software package for spectral simulation and analysis in EPR” *J. Magn. Reson.* **2006**, *178*, 42–55.

[17] M. Marchi, E. Raciti, S. M. Gali, F. Piccirilli, H. Vondracek, A. Actis, E. Salvadori, C. Rosso, A. Criado, C. D’Agostino, L. Forster, D. Lee, A. C. Foucher, R. K. Rai, D. Beljonne, E. A. Stach, M. Chiesa, R. Lazzaroni, G. Filippini, M. Prato, M. Melchionna, P. Fornasiero, “Carbon Vacancies Steer the Activity in Dual Ni Carbon Nitride Photocatalysis” *Adv. Sci.* **2023**, *10*, 2303781.

[18] J. P. Perdew, K. Burke, M. Ernzerhof, “Generalized Gradient Approximation Made Simple” *Phys. Rev. Lett.* **1996**, *77*, 3865–3868.

[19] S. Grimme, J. Antony, S. Ehrlich, H. Krieg, “A consistent and accurate *ab initio* parametrization of density functional dispersion correction (DFT-D) for the 94 elements H-Pu” *J. Chem. Phys.* **2010**, *132*, 154104.

[20] F. Weigend, R. Ahlrichs, “Balanced basis sets of split valence, triple zeta valence and quadruple zeta valence quality for H to Rn: Design and assessment of accuracy” *Phys. Chem. Chem. Phys.* **2005**, *7*, 3297–3305.

[21] S. Sinnecker, L. D. Slep, E. Bill, F. Neese, “Performance of Nonrelativistic and Quasi-Relativistic Hybrid DFT for the Prediction of Electric and Magnetic Hyperfine Parameters in 57Fe Mössbauer Spectra” *Inorg. Chem.* **2005**, *44*, 2245–2254.

[22] F. Neese, “Software update: The ORCA program system—Version 5.0” *WIREs Comput. Mol. Sci.* **2022**, *12*, e1606.

[23] N. Allasia, S. Xu, S. F. Jafri, E. Borfecchia, L. A. Cipriano, G. Terraneo, S. Tosoni, L. Mino, G. Di Liberto, G. Pacchioni, G. Vilé, “Resolving the Nanostructure of Carbon Nitride-Supported Single-Atom Catalysts” *Small* **2025**, *21*, 2408286.

[24] “Euler angles,” can be found under https://easyspin.org/easyspin/documentation/eulerangles.html(accessed 16 June 2025), **n.d.**
